# Identification and Characterization of CD8^+^CD27^+^CXCR3^−^ T Cell Dysregulation and Progression‐Associated Biomarkers in Systemic Lupus Erythematosus

**DOI:** 10.1002/advs.202300123

**Published:** 2023-10-24

**Authors:** Lulu Zhang, Fang Du, Qiqi Jin, Li Sun, Boqian Wang, Ziyang Tan, Xinyu Meng, Baozhen Huang, Yifan Zhan, Wenqiong Su, Rui Song, Chunmei Wu, Luonan Chen, Xiaoxiang Chen, Xianting Ding

**Affiliations:** ^1^ Department of Rheumatology Shanghai Jiao Tong University School of Medicine Affiliated Renji Hospital and School of Biomedical Engineering Shanghai 200030 China; ^2^ State Key Laboratory of Oncogenes and Related Genes Institute for Personalized Medicine School of Biomedical Engineering Shanghai Jiao Tong University Shanghai 200001 China; ^3^ Key Laboratory of Systems Biology Center for Excellence in Molecular Cell Science Shanghai Institute of Biochemistry and Cell Biology Chinese Academy of Sciences Shanghai 200031 China; ^4^ University of Chinese Academy of Sciences Beijing 100049 China; ^5^ School of Life Science and Technology ShanghaiTech University Shanghai 201210 China; ^6^ Department of Rheumatology and Immunology The First Affiliated Hospital of Wenzhou Medical University Wenzhou 325000 China; ^7^ Science for Life Laboratory Department of Women's and Children's Health Karolinska Institutet Solna 17121 Sweden; ^8^ Department of Chemical Pathology Li Ka Shing Institute of Health Sciences Faculty of Medicine The Chinese University of Hong Kong Hong Kong 999077 China; ^9^ Drug Discovery Shanghai Huaota Biopharmaceutical Co. Ltd. Shanghai 200131 China; ^10^ Nantong First People's Hospital Affiliated Hospital 2 of Nantong University Nantong Hospital of Renji Hospital Affiliated to Shanghai Jiao Tong University School of Medicine; ^11^ Key Laboratory of Systems Health Science of Zhejiang Province School of Life Science Hangzhou Institute for Advanced Study University of Chinese Academy of Sciences Chinese Academy of Sciences Hangzhou 310024 China

**Keywords:** dynamic network biomarker, mass cytometry, single‐cell RNA sequencing, systemic lupus erythematosus

## Abstract

Systemic Lupus Erythematosus (SLE) etiopathogenesis highlights the contributions of overproduction of CD4^+^ T cells and loss of immune tolerance. However, the involvement of CD8^+^ T cells in SLE pathology and disease progression remains unclear. Here, the comprehensive immune cell dysregulation in total 263 clinical peripheral blood samples composed of active SLE (aSLE), remission SLE (rSLE) and healthy controls (HCs) is investigated via mass cytometry, flow cytometry and single‐cell RNA sequencing. This is observed that CD8^+^CD27^+^CXCR3^−^ T cells are increased in rSLE compare to aSLE. Meanwhile, the effector function of CD8^+^CD27^+^CXCR3^−^ T cells are overactive in aSLE compare to HCs and rSLE, and are positively associated with clinical SLE activity. In addition, the response of peripheral blood mononuclear cells (PBMCs) is monitored to interleukin‐2 stimulation in aSLE and rSLE to construct dynamic network biomarker (DNB) model. It is demonstrated that DNB score‐related parameters can faithfully predict the remission of aSLE and the flares of rSLE. The abundance and functional dysregulation of CD8^+^CD27^+^CXCR3^−^ T cells can be potential biomarkers for SLE prognosis and concomitant diagnosis. The DNB score with accurate prediction to SLE disease progression can provide clinical treatment suggestions especially for drug dosage determination.

## Graphical abstract

This research examines the extensive immune cell dysregulation in systemic lupus erythematosus (SLE) by analyzing a total of 263 clinical samples through multi‐analyzer. In combination with dynamic network biomarker (DNB) score analysis, the study has identified disease‐progression associated CD8^+^CD27^+^CXCR3^−^ T cell signatures, with great potential in accurately predicting the progression of SLE and in providing crucial guidance on clinical treatment options.

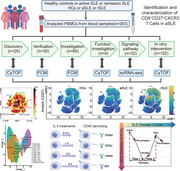

## 1 Introduction

Systemic lupus erythematosus (SLE) is an autoimmune disease characterized by tolerance loss to nuclear antigens, immune complexes deposition in tissues, and multi‐organ involvement. Despite frequent experience‐based revisions in diagnosis criteria and treatment regimens to provide consensus guidance, the SLE mortality rate remains high.^[^ ^1^, ^2^, ^3^ ^]^ The underlying immunopathogenesis of SLE involves the production of plasma circulating soluble factors such as autoantibodies and pro‐inflammatory cytokines, and activation of multiple innate and adaptive cell subsets,^[^ ^4^ ^]^ which has significantly improved prognosis of SLE patients.

Many cytokines (such as interferons (IFN), interleukin‐4 and interleukin‐17A) are upregulated in SLE patients to promote autoantibody production.^[^ ^4^ ^]^ Type I IFNs (particularly IFN‐α), produced by innate plasmacytoid dendritic cells (pDCs) in response to the endogenous nucleic acid via cytoplasmic nucleic acid sensors and endosomal Toll­like receptors, played the principal role in SLE immunopathogenesis.^[^ ^5^ ^]^ In contrast, type II IFN (IFN‐γ) is produced predominantly by CD4^+^ T helper 1, CD8^+^ T and natural killer (NK) cells, which also has an essential role in both spontaneous and induced lupus‐like disease.^[^ ^6^ ^]^

A hallmark of SLE immunopathology is the increased presence of autoantibody (essential mediators of SLE pathology)‐producing B cells.^[^ ^7^ ^]^ Whereas CD4^+^ T cells, especially T follicular helper cells, are recognized as the most efficient drivers of B cell differentiation into pathogenic autoantibody‐producing cells.^[^ ^4^ ^]^ Loss of T­cell tolerance due to deficient or defective regulatory T (Treg) cells has also been implied in SLE.^[^ ^8^ ^]^ Up‐regulation activated CD8^+^ cytotoxic T lymphocytes (CTLs) expressing perforin and granzyme B are correlated with SLE disease activity.^[^ ^9^ ^]^ CD8^+^ T cells with dampened cytotoxic function might lead to autoimmunity due to defective responses to viral antigens. As multiple mechanisms can lead to dysregulated T cell responses and T‐cell mediated autoimmunity, emerging evidence has linked autophagy (a conserved bulk‐degradation mechanism^[^ ^10^ ^]^) to SLE pathogenesis.^[^ ^11^, ^12^ ^]^ However, precise autophagy‐related dysregulation in specific cell subset in SLE remains unclear.

Herein, to address these knowledge gaps, we systematically depicted the complex landscape of immune cells associated with SLE immunopathogenesis in a well‐characterized SLE cohort consisting of clinically active (aSLE) and remission SLE patients (rSLE). This cohort covers across the whole SLE disease spectrum, including treatment naïve aSLE, treatment ongoing aSLE and rSLE with time‐lapse self‐comparison. Thus, the examination of immune cell cluster abundance and functional protein dysregulations across this cohort may offer enhanced understanding of the functional and phenotypic features of SLE and provide potential diagnosis and treatments suggestions for SLE patients.

## 2 Results

### 2.1 Overview of Study Design

**Figure 1:**
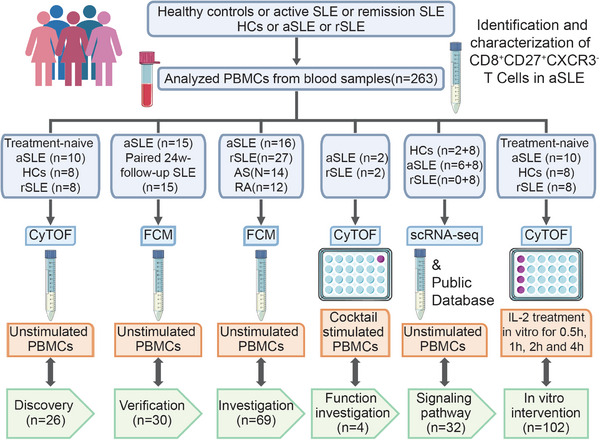
Overview of study design. AS, ankylosing spondylitis; aSLE, active SLE patients; CyTOF, mass cytometry; FCM, flow cytometry; HCs, healthy controls; IL‐2, interleukin‐2; PBMCs, peripheral blood mononuclear cells; RA, rheumatoid arthritis; rSLE, remission SLE; scRNA‐seq, single‐cell RNA sequencing; SLE, systemic lupus erythematosus. The Cocktail stimulated samples indicated SLE patients are stimulated with the cocktail of PMA, Ionomycin and Brefeldin A. All the measurement and analyzed samples were PBMCs collected from fresh blood samples.

**Figure** **1** provides an overview of the research design. High‐dimensional mass cytometry (CyTOF) was used to systematically describe immune dysfunction in SLE using paired aSLE and rSLE samples. Then to verify the discoveries from CyTOF results, we collected 15 paired aSLE and 24‐week follow‐up rSLE blood samples and analyzed them using flow cytometry (FCM). To further verify and determine whether our findings could apply to non‐paired SLE or other autoimmune disease samples, we analyzed 69 additional blood samples via FCM, including from rheumatoid arthritis (RA) and ankylosing spondylitis (AS) patients.

For functional analysis, we analyzed peripheral blood mononuclear cells (PBMCs) responses to a cocktail of phorbol 12‐myristate 13‐acetate (PMA), ionomycin and brefeldin A, and performed single‐cell RNA sequencing (scRNA‐seq) analysis. In addition, we also analyzed scRNA‐seq data of SLE patients from public database for a deep understanding of the dysregulated immune phenotyping in SLE. Also for further dynamic functional investigation, the response of PBMCs to IL‐2 stimulation were monitored.

### 2.2 Remission SLE Patients Demonstrate Elevated CD8^+^CD27^+^CXCR3^−^ T Cell Frequency

To comprehensively profile the complexity of immune cell dysregulation across the disease spectrum and further investigate CD8^+^ T cells involvement in SLE diseased progression, we further analyzed 29 functional protein biomarkers in HCs, treatment‐naïve aSLE and rSLE by CyTOF (Tables S1,S2,S3 and Figure S1, Supporting Information).

**Figure 2:**
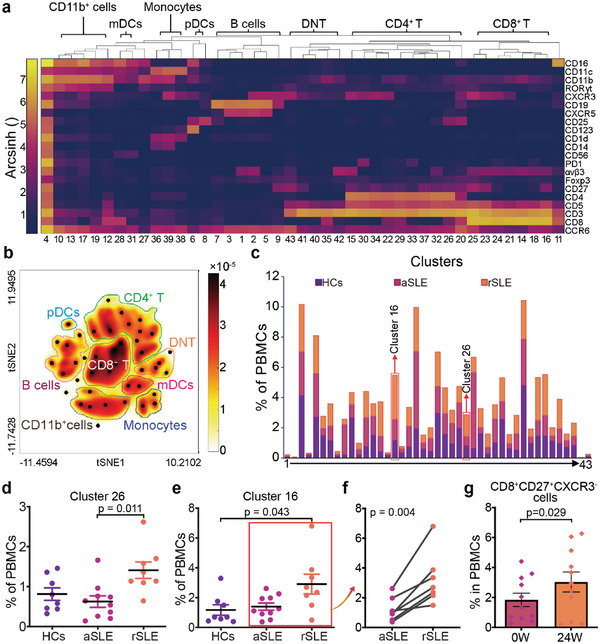
rSLE patients demonstrate elevated CD8^+^CD27^+^CXCR3^−^ T cell frequency. a) Combined biomarker expression patterns of 43 cell clusters. b) The 43 cell clusters were annotated as traditional cell lineages based on combined biomarker expression patterns. c) Mean cell frequencies (color bars) of 43 cell clusters among total PBMCs (x‐axis) (HCs (*n* = 8), treatment‐naïve aSLE (*n* = 10) and rSLE (*n* = 8)). d,e) Individual d) Cluster 26 (CD4^+^CD25^+^Foxp3^−^) and e) Cluster 16 (CD8^+^CD27^+^CXCR3^−^) T cell abundance among total PBMCs across 3 groups. In the box plot, the data are presented as Mean±SEM and the p value is based on Kruskal‐Wallis test, post‐test p < 0.05. f) The paired sample t‐test of Cluster 16 cell abundance among total PBMCs between self‐matched treatment‐naïve aSLE (*n* = 7) and rSLE (*n* = 7). For the paired sample t‐test, p value is based on two‐tailed Wilcoxon signed‐rank tests. g) The paired sample t‐test result of CD8^+^CD27^+^CXCR3^−^ T cell abundance among total PBMCs between another 11 self‐matched aSLE and rSLE measured via FCM. The p value is based on a two‐tailed paired test. aSLE, active SLE patients; CyTOF, mass cytometry; DNT, double‐negative T cells; HCs, healthy controls; mDCs, myeloid dendritic cells; pDCs, plasmacytoid dendritic cells; rSLE, remission SLE patients.

For high‐dimensional CyTOF data, after pre‐processing and manual gating of CD45^+^ cells,^[^ ^13^ ^]^ the PBMCs were automatically partitioned into 43 cell clusters (Figure S2a,b, Supporting Information) with Automatic Classification of Cellular Expression by Nonlinear Stochastic Embedding (ACCENSE)^[^ ^14^ ^]^ based on expression levels of lineage‐associated markers (Table S3, Supporting Information). The data were visualized on a t‐distributed stochastic neighbor embedding (t‐SNE) map^[^ ^15^ ^]^ for an overview of identified leukocyte clusters (Figure S2c, Supporting Information). According to their combined expression patterns (**Figure** **2**a), these 43 clusters were manually annotated into 7 major immune cell lineages: CD4^+^ T (10 clusters :15, 20, 22, 26, 29, 30, 32, 33, 34, and 37), CD8^+^ T (8 clusters: 11, 14, 16, 18, 21, 23, 24, and 25), double‐negative T cells (5 clusters: 35, 40, 41, 42, and 43), pDCs (Cluster 6 and 8), myeloid dendritic cells (Cluster 28 and 31), monocytes (Cluster 36, 38, and 39), and B cells (Cluster 1, 2, 5, 3, 7, and 9) (Figure 2b). The detailed cell subtype for each cluster were shown in Table S4 (Supporting Information).

The cell‐type composition of PBMCs across three groups (HCs, aSLE and rSLE) varied substantially (Figure 2c and Supplementary Figure S3a). We observed decreased cell frequencies of Treg cells (Cluster 26, CD4^+^CD25^+^FoxP3^+^ T cells) in treatment‐naïve aSLE compared with HCs and rSLE (Supplementary Figure S3b,c), which is consistent with previous literature report.^[^ ^16^ ^]^

Besides, the statistical analysis revealed that cell frequency of Cluster 16 (CD8^+^ CD27^+^ CXCR3^−^ T cells), the phenotype of which are similar with classic CD8^+^ memory effector T cells,^[^ ^17^ ^]^ in rSLE were 2.52 times higher than that of HCs (p = 0.043) (Figure 2e; Figure S4a, Supporting Information). Moreover, in 7 self‐matched treatment‐naïve aSLE and rSLE, clinical medical treatment significantly increased the frequencies of CD8^+^CD27^+^CXCR3^−^ T cells (p = 0.004, Figure 2f). CD8^+^ T cells can be classified from naive, effector and different memory subsets^[^ ^18^ ^]^ and CD27 is a memory marker.^[^ ^19^ ^]^ CD8^+^ T cells with memory phenotype correlates with poor prognosis and increase with disease durations.^[^ ^20^ ^]^ For CD8^+^ T cells, automated clustering further divided CD8^+^CD27^+^ T cells into 3 clusters. For cross‐verification, we further calculated the cell frequency of Clusters 16, 23 and 25 in total CD8^+^CD27^+^ T cells. Only CD8^+^CD27^+^CXCR3^−^ T cell frequencies in rSLE increased significantly (Figure S4, Supporting Information). Besides, manually gated cell frequencies of Cluster 16 in rSLE were also statistically higher than HCs and treatment‐naïve aSLE (Figure S5, Supporting Information). These results indicated that the elevated frequency of CD8^+^ CD27^+^ CXCR3^−^ T cells in rSLE is the unique feature of SLE.

To further verify the dysregulation of CD8^+^CD27^+^CXCR3^−^ T cells in CyTOF dataset, we recruited another 15 aSLE patients (Figure S6a, Supporting Information) for cross‐validation. After 6 months follow‐up, 11 SLE patients turned into remission while 4 still progressed. We measured the frequency of CD8^+^CD27^+^CXCR3^−^ T cells (Figure S6b, Supporting Information), and our results further confirmed the elevated CD8^+^CD27^+^CXCR3^−^ T cells frequency were consistently observed for all the 11 rSLE, while the 4 aSLE still in progression conversely all showed decreased cell frequency (Figure 2g; Figure S6c, Supporting Information). In addition, we analyzed the frequency of CD8^+^CD27^+^CXCR3^−^ T cells from another unpaired SLE samples including 10 aSLE and 15 rSLE, similarly showing the significant increase in the rSLE samples, compared with aSLE samples, which further verified our discovery (Supplementary Figure S6d).

We then further recruited 6 aSLE, 12 non‐paired rSLE, 14 RA and 12 AS patients to examine whether this dysregulation is universally observed in other autoimmune diseases (Figure S6e, Supporting Information). The frequency of CD8^+^CD27^+^CXCR3^−^ T cells was also elevated in patients with rSLE compared with aSLE, although the difference was not statistically significant, which could be attributed to the high heterogeneity in SLE patients. In addition, the frequency of CD8^+^CD27^+^CXCR3^−^ T cells in rSLE was significantly higher than that in RA and AS. These results provided confirmatory evidence that an elevated frequency of CD8^+^CD27^+^CXCR3^−^ T cells is a unique characteristic of patients with rSLE. Therefore, closely monitoring the abundance of CD8+CD27+CXCR3^−^ T cells could serve as a potential diagnostic biomarker to concomitantly guide clinical management of aSLE.

### 2.3 CD8^+^CD27^+^CXCR3^−^ T Cells in Treatment‐Naïve aSLE are Functionally Overactive

**Figure 3:**
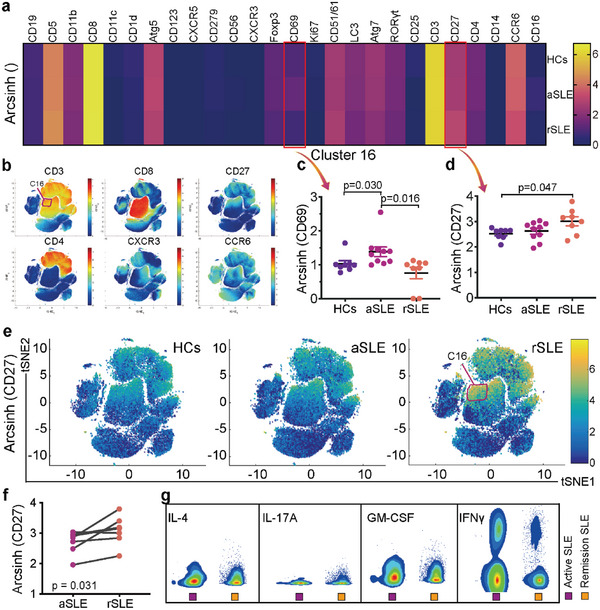
CD8^+^CD27^+^CXCR3^−^ T cells in treatment‐naïve aSLE are functionally overactive. a) Biomarker abundance in Cluster16 cells. b) The t‐SNE plots showed expressions of representative biomarkers in CD8^+^CD27^+^CXCR3^−^ T cells. Individual expressions of c) CD69 and d) CD27 across HCs (n = 8), aSLE (n = 10) and rSLE (n = 8). In box plots, data are presented as Mean±SEM and p values are based on Kruskal‐Wallis test, post‐test p < 0.05. e) Single‐cell t‐SNE profiling of CD27. The red squares outline the Cluster 16 cells. f) The paired t‐test of CD27 abundance between self‐matched treatment‐naïve aSLE (n = 7) and rSLE (n = 7). For the paired sample t‐test, p value is based on two‐tailed Wilcoxon signed‐rank tests. g) Indicated cytokine abundance of Cluster 16 cells from representative aSLE (purple boxes) and rSLE (orange boxes). Their PBMCs were stimulated with the cocktail of PMA, Ionomycin and Brefeldin A, measured via CyTOF and gated manually. aSLE, active SLE patients; HCs, healthy controls; rSLE, remission SLE patients.

To investigate whether CD8^+^CD27^+^CXCR3^−^ T cells exhibit molecular characteristics indicative of effector function,^[^ ^21^ ^]^ we further analyzed the expression of biomarkers associated with cell activation and function. CD69 and CD27 expressions varied distinctly across HCs, treatment‐naïve aSLE and rSLE (**Figure** **3**a,b; Figure S7, Supporting Information).

Expression of CD69 (a marker for early cell activation^[^ ^22^ ^]^) in treatment‐naïve aSLE was ≈2 times higher than that in rSLE (p = 0.016) (Figure 3c). Expression of CD27 (a biomarker of memory T cells and low expression in terminally differentiated effector T cells^[^ ^23^ ^]^) in rSLE was 1.35 times higher than that in HCs (p = 0.047) (Figure 3d,e). The paired t‐test between self‐matched treatment‐naïve aSLE and rSLE shows that CD27 expressions in rSLE were statistically higher than those in treatment‐naïve aSLE (p = 0.031) (Figure 3f). These results suggest that CD8^+^CD27^+^CXCR3^−^ T cells were functionally more active in treatment‐naïve aSLE as compared to rSLE.

We then further investigated the effector functions of CD8^+^CD27^+^CXCR3^−^ T cells in SLE. PBMCs from treatment‐naïve aSLE and rSLE were stimulated with the Leukocyte Activation Cocktail (see Methods and materials) and analyzed by CyTOF. Our results showed that CD8^+^CD27^+^CXCR3^−^ T cells mainly produced IFNγ (Figure 3g; Figure S8a, Supporting Information). Although CD8^+^CD27^+^CXCR3^−^ T cells from both treatment‐naïve aSLE and rSLE secreted IFNγ, frequencies of IFNγ^+^ cells in rSLE were lower than treatment‐naïve aSLE (Figure S8b–d, Supporting Information). Notably, CD8^+^CD27^+^CXCR3^−^ T cell abundance in rSLE was also higher than that in treatment‐naïve aSLE, which is again consistent with our previous finding (Figure 2e).

### 2.4 The scRNA‐seq Results of HCs and aSLEs Indicate CD8^+^CD27^+^CXCR3^−^ T Cells with Specific Effector Function and Were Overactive in aSLE

**Figure 4:**
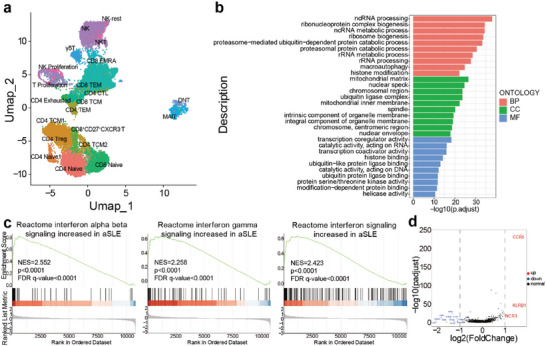
The scRNA‐seq analysis indicates the CD8^+^CD27^+^CXCR3^−^ T cells upregulated IFNγ secretion in aSLE. a) The UMAP showed the immune cell subsets from scRNA‐seq clustering. b) The Biological processes (BP), cellular component (CC) and molecular function (MF) of gene ontology (GO) in total differentially expressed genes (DEGs) of CD8^+^CD27^+^CXCR3^−^ T cells. c) The Gene Set Enrichment Analysis (GSEA) result showed upregulated interferon signaling pathway of CD8^+^CD27^+^CXCR3^−^ T cells in aSLE compared with HCs. d) The volcano result showed the genes which increased or decreased significantly in CD8^+^CD27^+^CXCR3^−^ T cells.

Based on above results, we hypothesized the over‐expression of IFNγ in CD8^+^CD27^+^CXCR3^−^ T cells might contributed to SLE pathogenesis. We therefore further performed scRNA‐seq experiment for PBMCs from another 6 aSLE and 2HCs (Figure S9, Supporting Information). PBMCs were divided into different subsets according their function as shown in the Umap (**Figure** **4**a). Based on the position of CD8^+^CD27^+^CXCR3^−^ T cells, these cells can be defined as effector memory‐like T cells.

According to the 1822 differently expressed genes (DEGs) of CD8^+^CD27^+^CXCR3^−^ T cells in aSLE compared with HCs, we first implemented gene ontology (GO) analysis (Figure 4b). In aSLE, the DEGs were enriched in macroautophagy and ubiquitin ligase complex biological processes (BPs), ubiquitin ligase complex and integral component of organelle membrane cellular components (CCs), and ubiquitin−like protein ligase binding and ubiquitin protein ligase binding molecular functions (MFs), which are all highly associated with macroautophagy activity.^[^ ^24^, ^25^ ^]^ This result indicated that CD8^+^CD27^+^CXCR3^−^ T cells might showed upregulated autophagy activity and was consistent with the increased means of autophagy related proteins in aSLE.

Further signaling pathway results via the Gene Set Enrichment Analysis (GSEA) showed that the major pathway differences between aSLE and HCs focused on interferon signaling pathway (Figure 4c; Figure S10, Supporting Information), which was consistent with the high expression of IFNγ in aSLE (Figure 3g) and further indicated the overactive effector function in aSLE (Figure 4b). When focused on specific genes, we noticed that there were only 3 majorly upregulated genes in aSLE (Figure 4d). NCR3, natural cytotoxicity triggering receptor 3, also known as NKp30, can be triggered via specific ligands and involve IFNγ serection.^[^ ^26^ ^]^ KLRB1, which encodes the NK cell receptor NKR‐P1, possesses active function.^[^ ^27^ ^]^ These results suggest that the CD8^+^CD27^+^CXCR3^−^ T cells might have certain effector function and were overactive in aSLE.

### 2.5 The scRNA‐seq Results of HCs, aSLEs, and rSLEs from Public Database Verify the Overactive Effector Functions of CD8^+^CD27^+^CXCR3^−^ T Cells in aSLE

**Figure 5:**
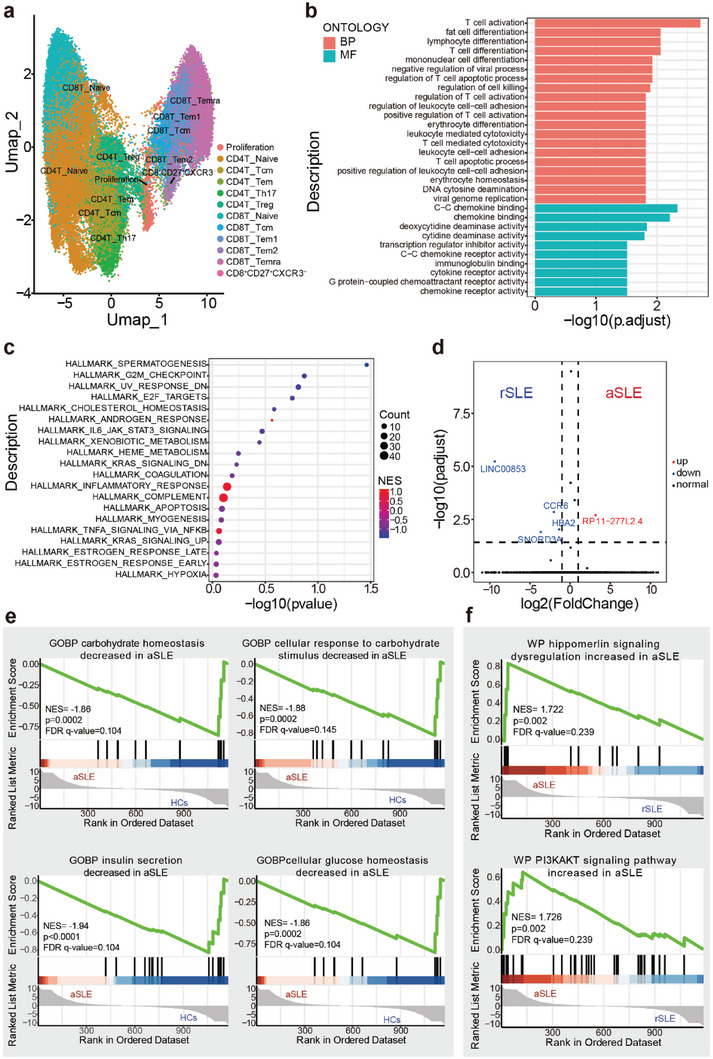
The scRNA‐seq analysis results from published data verify the overactive effector functions of CD8^+^CD27^+^CXCR3^−^ T cells in aSLE. a) The UMAP showed the immune cell subsets from scRNA‐seq clustering of CD3^+^ T cellss. b) The Biological processes (BP), cellular component (CC) and molecular function (MF) of gene ontology (GO) in total differentially expressed genes (DEGs) of CD8^+^CD27^+^CXCR3^−^ T cells. c) The Gene Set Enrichment Analysis (GSEA) result showed hallmark signaling pathway of CD8^+^CD27^+^CXCR3^−^ T cells. d) The volcano result showed the genes which increased or decreased significantly of CD8^+^CD27^+^CXCR3^−^ T cells in rSLE compared with HCs. e) Glucose response related pathway were significantly decreased in aSLE compared with HCs. f) The WP result indicated the genes of PI3KAKT signaling pathway and hippomerlin signaling dysregulation positively enriched in aSLE compared with rSLEs.

Considering the lack of rSLE scRNA‐seq data in our results, we then analyzed scRNA‐seq data of 8 HCs, 8 aSLEs and from public database.^[^ ^28^ ^]^ We totally analyzed 48456 T cells of these 24 samples and the cluster results in Umap (**Figure** **5**a) and The pseudo‐time series analysis (Figure S11, Supporting Information) both indicated the CD8^+^CD27^+^CXCR3^−^ T cells could be classified into effector memory T cells.

We then analyzed the DEGs of CD8^+^CD27^+^CXCR3^−^ T and found the upregulated genes were similar results with our performed scRNA‐seq results (Figure 4; Figure S12a,b, Supporting Information). For signaling pathway analysis, the significant upregulated of genes in T cell activation and cell differentiation BPs (Figure 5b), inflammatory response and complement signaling pathways (Figure 5c) also indicated the effector function of CD8^+^CD27^+^CXCR3^−^ T cells.

To further understanding the immune deregulation of CD8^+^CD27^+^CXCR3^−^ T cells in aSLE, we compared the DEGs between each 2 groups (Figure 5d; Figure S12c,d, Supporting Information) and found the significant upregulated RP11‐277L2.4 expressions in aSLE compared with both HCs and rSLE, while no significant differences between HCs and rSLE.

Regarding the differences in signaling pathway, the aSLE patients showed dysregulated carbohydrate homeostasis and tolerance to high‐glucose level, this might be part reason of the elevated autophagy biomarker expression levels in aSLE due to nutrition lack is a major cause of autophagy behavior.^[^ ^29^ ^]^ When compared with rSLE, aSLE showed positively enriched genes in hippoemerlin signaling dysregulation and PI3K/AKT signaling pathway. According to previous studies, dysregulation of the PI3K/AKT pathway contributes to the development of autoimmunity and SLE and lead to the production of pro‐inflammatory cytokines like IFNγ.^[^ ^30^ ^]^ Hippoemerlin signaling is tumor related signaling pathway and play key roles promoting cell proliferation. The dysregulation related genes enriched in aSLE could also indicate an active immune status and could also be reasons of elevated proportion of CD8+CD27+CXCR3^−^ T cells in rSLE.^[^ ^31^ ^]^

### 2.6 SLE Clinical Disease Activity is Positively Associated with the Effector Function and Autophagy Activity of CD8^+^CD27^+^CXCR3^−^ T Cells

**Figure 6:**
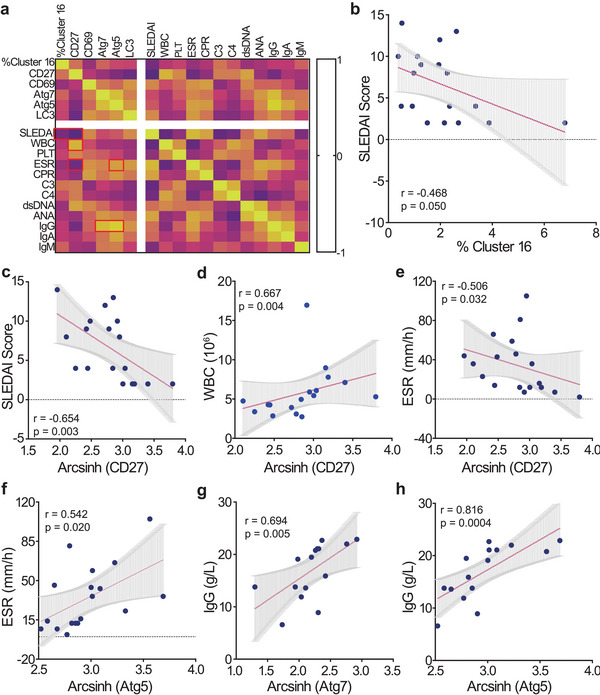
SLE clinical disease activity is positively associated with the effector function and autophagy activity of CD8^+^CD27^+^CXCR3^−^ T cells. a) Clinical characteristics and molecular signatures of CD8^+^CD27^+^CXCR3^−^ T cells are pooled for correlation analysis. Spearman's correlation was performed on any two of these parameters. All r values of Spearman's correlation coefficient are shown in the heatmap. The color bar represents the value of the correlation values. Correlation analysis between SLEDAI score and b) cell frequencies and c) arcsinh (CD27). d) Correlation analysis between WBC and arcsinh (CD27). Correlation analysis between ESR and e) arcsinh (CD27) and f) arcsinh (Atg7). Correlation analysis between IgG and g) arcsinh (Atg7) and h) arcsinh (Atg5). All r and p values are based on Spearman's correlation coefficient. ANA, antinuclear antibodies; C3, complement 3; C4, complement 4; CPR, C‐reactive protein; ELISA, enzyme‐linked immunosorbent assay; ESR, erythrocyte sedimentation rate; PLT, platelets; SLEDAI, SLE disease activity index; WBC, white blood cells.

Based on the above findings, we hypothesized that the elevated effector function and autophagy activity of CD8^+^CD27^+^CXCR3^−^ T cells are associated with clinical disease activity in SLE patients. To verify our hypothesis, we analyzed SLE clinical disease activity versus the effector function and autophagy activity of CD8^+^CD27^+^CXCR3^−^ T cells (**Figure** **6**a; Figure S13, Supporting Information). The SLEDAI score was negatively associated with CD8^+^CD27^+^CXCR3^−^ T cell frequencies and CD27 expressions (Figure 6b,c). CD27 expression was positively associated with numbers of leukocytes (Figure 6d). These results verified the hypothesis that elevated cell frequencies and CD27 expressions of CD8^+^CD27^+^CXCR3^−^ T cells contribute to immune tolerance in SLE.

Erythrocyte sedimentation rate (ESR) is a routine clinical testing indicator for SLE and regularly elevates in active SLE patients.^[^ ^32^ ^]^ We found that ESR values were negatively associated with CD27 expressions and positively associated with abundances of CD69, Atg5 and LC3 (Figure 6e,f; Figure S14a,b, Supporting Information). We found that C‐reactive protein abundance (CRP, a biomarker for inflammation^[^ ^33^ ^]^) was also positively associated with CD69 and Atg5 expressions (Figure S14c,d, Supporting Information).

Antinuclear antibodies (ANA) are critical clinical diagnosis biomarkers for SLE.^[^ ^3^ ^1]^ Our analysis revealed that ANA abundance was positively associated with Atg7 (Figure S14e, Supporting Information). IgG^[^ ^34^ ^]^ level was also positively associated with Atg7 and Atg5 expressions (Figure 6g,h). Besides, IgA level was positively associated with Atg5 expressions (Figure S14f, Supporting Information).

As multiple mechanisms can lead to dysregulated T cell responses and T cell mediated autoimmunity, emerging evidence has linked autophagy (a conserved bulk‐degradation mechanism) to SLE pathogenesis.^[^ ^11^ ^]^ Autophagy can regulate IFN production, inflammasome activation and promote MHC presentation to affect diverse aspects of the immune system.^[^ ^29^, ^35^ ^]^ These results indicate that augmented effector function and autophagy activity of CD8^+^CD27^+^CXCR3^−^ T cells in treatment‐naïve active SLE might exacerbate the disease severity. Besides, our data suggest that CD8^+^CD27^+^CXCR3^−^ T cells could be a potential biomarker to monitor disease activity and predict clinical treatment progression.

### 2.7 IL‐2 Treatment Rescues Phenotypes of CD8^+^CD27^+^CXCR3^−^ T Cells

**Figure 7:**
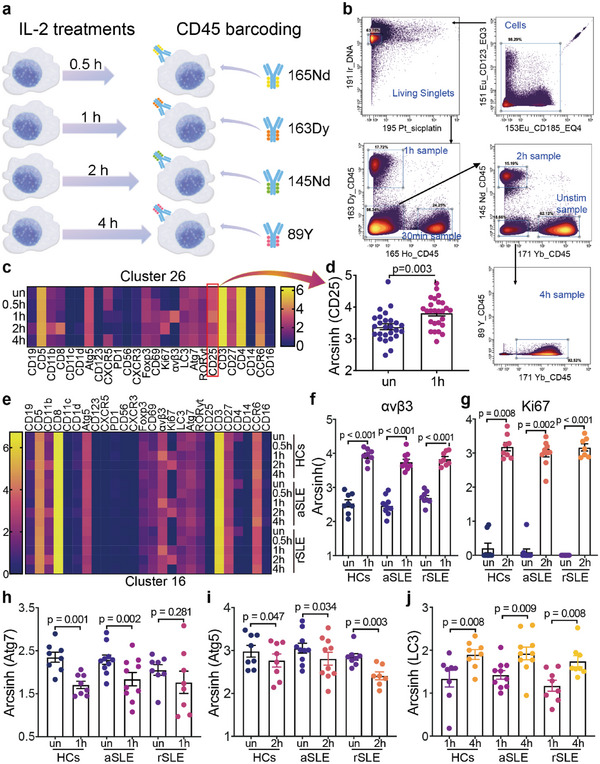
IL‐2 treatment rescues phenotypes of Cluster 16 (CD8^+^CD27^+^CXCR3^−^)T cells. a) PBMCs of HCs, aSLE and rSLE patients were treated with IL‐2 for 0.5, 1, 2, and 4 h, respectively. Anti‐CD45 barcoding strategy before commercial Pd barcoding was applied. b) Debarcoding strategy for anti‐CD45 barcoding pipeline. c) Protein biomarker expressions of Cluster 26 (CD4^+^CD25^+^Foxp3^+^, Treg cells) under different IL‐2 treatments. The color bar represents the value of the biomarker expression levels. d) Individual CD25 expressions of Cluster 26 from unstimulated and IL‐2 treatment PBMCs. The p value is based on two‐tailed paired t test. e) Protein biomarker expression of Cluster 16 across 3 groups from PBMCs treated with IL‐2 for 0, 0.5, 1, 2, and 4 h. The color bar represents the value of the biomarker expression levels. f) Integrin αvβ3 of Cluster 16 expression showed a uniform increase after IL‐2 treatment for 1 h. The p values are based on the paired t tests. g) Ki67 expression showed a significant increase after IL‐2 treatment for 2 h. The p value of HCs and treatment‐naïve aSLE is based on the Wilcoxon matched‐pairs signed‐rank tests and rSLE is based on Mann‐Whitney test. Expressions of h) Atg7 and i) Atg5 from unstimulated and IL‐2 treatment conditions. j) LC3 expressions after IL‐2 treatment for 1 and 4 h. Data are presented as Mean±SEM. p value is based on Turkey's multiple comparison test. IL‐2, interleukin‐2; un, unstimulated; 0.5 h, PBMCs treated with IL‐2 for 0.5 h; 1 h, PBMCs treated with IL‐2 for 1 h; 2 h, PBMCs treated with IL‐2 for 2 h; 4 h, PBMCs treated with IL‐2 for 4 h.

IL‐2 is a pleiotropic cytokine. Low‐dose IL‐2 has been proved to be an effective, well‐tolerated and safe regimen for clinical SLE treatment via selectively expanding and restoring Treg cells.^[^ ^36^ ^]^ Notably, IL‐2 could determine the fate of both pro‐ and anti‐inflammatory immune cells by regulating transcriptional and metabolic programs.^[^ ^37^, ^38^ ^]^ For example, via Janus family kinase (JAK) members, IL‐2 could control the expression of effector CTLs like IFNγ^[^ ^37^ ^]^ and activate mammalian target of rapamycin complex 1 (mTORC1) signal, which is also correlated with autophagy activity.^[^ ^38^, ^39^ ^]^ Besides, dynamically significant differences between healthy and morbid rather than static ones are more comprehensive to evaluate disease‐related dysfunction.^[^ ^40^ ^]^ Therefore, we treated PBMCs with low‐dose IL‐2 in vitro for 30 min, 1, 2, and 4 h, respectively (**Figure** **7**a), to investigate the dynamic differences between HCs and SLE patients of CD8^+^ T cells response to low‐dose IL‐2 effects.

To remove batch effects, we adopted a 2‐step barcoding strategy that could barcode over 100 samples simultaneously. Briefly, we utilized CD45 antibodies with different metal tags to barcode samples from a single person before the commercial Pd barcoding Kit.^[^^39]^ The debarcoding strategy of anti‐CD45 is provided in Figure 7b.

To prove the efficacy of IL‐2 treatment, we first evaluated IL‐2 effects on Treg cells. CD25 (also known as the α‐chain of the IL‐2 receptor, IL‐2Rα^[^ ^16^ ^]^) expression of Treg cells was upregulated significantly after IL‐2 treatment (Figure 7c,d; Figure S15a, Supporting Information). CD25 expression of manually gated CD4^+^CD25^+^ T cells also increased after IL‐2 treatment (Figure S14b, Supporting Information). Meanwhile, Foxp3 expression of Treg cells was upregulated after IL‐2 treatment, which indicated an amplified suppressive capacity of Treg cells^[^ ^16^ ^]^ (Figure S15c, Supporting Information). These results are consistent with documented research that IL‐2 could upregulate cell abundance and immune‐suppressive capacity of Treg cells.^[^ ^16^ ^]^

We further examined dynamic changes of all 25 protein biomarker expressions in CD8^+^CD27^+^CXCR3^−^ T cells (Figure 7e; Figure S16a, Supporting Information). As the heatmap shows, CD8^+^CD27^+^CXCR3^−^ T cells displayed upregulated expression of Integrin αvβ3 and Ki67 (Figure 7f,g). Moreover, the increase in CD51/61 (also known as integrin αvβ3) and Ki67 (a marker for cell proliferation^[^ ^42^ ^]^) showed no significant differences across HCs, aSLE and rSLE (Figure S16b–e, Supporting Information).

We then investigated the effects of IL‐2 treatment on CD69 and CD27. The CD69 expression from rSLE and HCs but not from treatment‐naïve aSLE was upregulated after IL‐2 treatment (Figure S17a, Supporting Information). Besides, IL‐2 treatment eliminated dysregulation in CD69 expression between self‐matched treatment‐naïve aSLE and rSLE (Figure S17b, Supporting Information). Meanwhile, IL‐2 treatment induced no significant effects on CD27 expressions (Figure S17c, Supporting Information). CD27 expression in rSLE was still significantly higher than that in HCs (p = 0.006) and treatment‐naïve aSLE (p = 0.008) after treated with IL‐2 for 4 h (Figure S17d, Supporting Information). These data indicate that IL‐2 treatment induced uniform and unique effects on the proliferation activity of CD8^+^CD27^+^CXCR3^−^ T cells.

Considering mTORC1 signaling is involved in both IL‐2 signaling pathway and autophagy activity,^[^ ^43^ ^]^ we then investigated whether IL‐2 would affect the autophagy activity of CD8^+^CD27^+^CXCR3^−^ T cells. We observed that IL‐2 treatment reduced expressions of Atg7, especially after treated with IL‐2 for 1 h (Figure 7h; Figure S18a, Supporting Information). Although when comparing all IL‐2 treatment conditions, Atg5 showed no statistical changes, paired t test showed Atg5 significantly decreased after treated with IL‐2 for 2 h (Figure 7i; Figure S18b, Supporting Information). In comparison with the variation of LC3 expressions in the early IL‐2 treatment stage, LC3 expression upregulates steadily by IL‐2 treatment from 1 to 4 h (Figure 7j; Figure S18c, Supporting Information). Considering that both Atg7 and Atg5 are necessary for autophagosome formation,^[^ ^29^ ^]^ which finally leads to LC3 degradation, the accumulation of LC3 from IL‐2 treatment for 1 h might indicate IL‐2 could reduce autophagy activity of CD8^+^CD27^+^CXCR3^−^ T cells. This observation was universal across HCs, treatment‐naïve aSLE and rSLE.

### 2.8 Long‐Term Follow‐Up Data Proved Dynamic Network Biomarker (DNB) Analysis Could Predict SLE Remission and Flares

Although advanced treatments are beneficial to SLE remission, most SLE patients would experience disease exacerbations (flares) and follow a typical relapsing‐remitting course.^[^ ^44^ ^]^ Identifying biomarkers to predict the risk of SLE relapse is critical to prognostic care in clinic. Dynamic network biomarker (DNB) is a new concept developed based on non‐linear dynamical and complex network theories, which could distinguish the pre‐disease state existed between healthy and pathologic states.^[^ ^40^, ^45^, ^46^ ^]^ DNB could quantify dynamical changes of molecule expressions and correlations between molecules of high‐dimensional datasets containing time series.^[^ ^46^, ^47^ ^]^ Unlike traditional biomarkers, DNB is a group of highly correlated biomarkers where their expressions change dynamically during the pre‐disease state.^[^ ^45^ ^]^ Herein, we applied DNB to determine potential flare biomarkers in SLE by evaluating the fluctuated protein expressions during IL‐2 treatment.

We first calculated the most correlated DNB biomarkers in SLE (Figure S19 Supporting Information). To explore the main functions of DNB members, we constructed protein‐protein association network with CD14, CD1d (CD1D), Ki67 (MKI67), and CD11c (ITGAX), and extended it from 4 to 14 proteins using STRING^[^ ^48^ ^]^ (Figure S20a, Supporting Information). The 14 proteins were enriched in Toll‐like receptor signaling pathway, Cellular response to lipopolysaccharide, Leukocyte activation and Innate immune response, which are all highly associated with immune system processes. In addition, Regulation of cellular component organization pathway was also enriched, which may imply changes in the organization and structure of cellular components in response to the IL‐2 treatment (Figure S20b, Supporting Information). We found that CD14 is involved in Toll‐like receptor 4 signaling pathway, CD14 and Ki67 (MKI67) are involved in Regulation of cellular component organization, leukocyte activation and Immune system process include the participation of CD14, CD1d (CD1D), and CD11c (ITGAX) (Figure S20c, Supporting Information). We also analyzed the protein‐protein network using 10 DNB members with a frequency of 5 or greater in SLE samples (Figure S20d, Supporting Information). Enrichment analysis of these 10 DNB members revealed their involvement in immune‐ and cytokine‐related pathways, as well as their close association with leukemia and lymphatic system diseases (Figure S20e, Supporting Information). In summary, our findings indicate that four DNB core proteins are involved in multiple immune‐related pathways, and the top 10 DNB members with high frequency in SLE samples play important roles in the occurrence and development of diseases such as leukemia and lymphatic system diseases.

**Figure 8:**
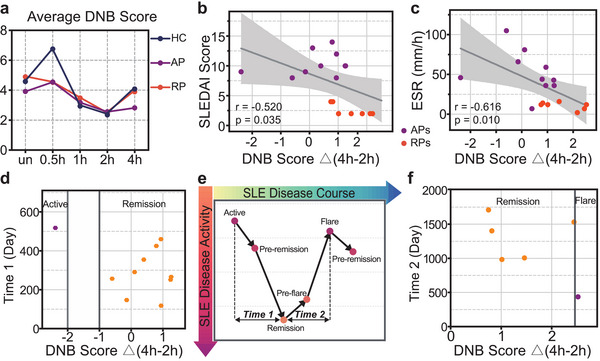
Long‐term follow‐up data proved DNB analysis could predict SLE remission and flares. a) The average DNB score across HCs, aSLE, and rSLE.The difference in DNB scores between 2 and 4 h IL‐2 treatment is negatively correlated with b) SLEDAI score and c) ESR. d) The difference in DNB scores between 2 and 4 h IL‐2 treatment faithfully distinguishes the remission and progression SLE patients during long‐term follow‐up. e) The schematic of SLE disease course with time‐lapse. f) The difference in DNB scores between 2 and 4 h IL‐2 treatment distinguishes the remission and flare SLE patients. The color in (d,e) represents the final disease activity after long‐time follow‐up. DNB, dynamic network biomarkers; ESR, erythrocyte sedimentation rate; SLEDAI, SLE disease activity index.

Based on the top DNB biomarkers, the DNB score variation after IL‐2 treatment for each patient was acquired (Figure S21a–c, Supporting Information). The HCs showed almost consistent change tendency while the SLE patients displayed distinct tendency. The average DNB score across HCs, aSLE and rSLE showed the aSLE has the tiniest variations after IL‐2 treatment (**Figure** **8**a). The correlation analysis showed that DNB score variation related with 2 h IL‐2 treatment was highly correlated with clinical features in SLE (Figures S21d,e and S22, Supporting Information). Particularly, the difference of DNB scores between 2 h and 4 h IL‐2 treatment was negatively correlated with SLEDAI score and ESR (Figure 8b,c).

We further investigated the relationship between DNB scores and disease progression. As shown in Figure 8d, most of the patients went through the remission and flare cycle over time. Based on long‐term follow‐up record for each patient, we compared the differences of DNB scores between 2 and 4 h IL‐2 treatment with patient disease time course. DNB related parameter could faithfully indicate whether the aSLE would get remission in the near future (Figure 8). Besides, DNB related parameter could also indicate whether the rSLE would flare (Figure 8f). These results proved DNB scores could readily serve as effective prognosis indicators for SLE remission and flares.

## 3 Discussion

The pathogenesis of SLE is commonly reported to associate with multiple CD4^+^ T cell subsets and related autophagy dysregulation.^[^ ^4^ ^]^ However, little evidence was previously available to explore the potential association between SLE and CD8^+^ T cells. Herein, we systematically depict the immune landscape in a well‐characterized SLE cohort, including newly diagnosed, treatment‐naïve patients and the follow‐up remission samples. The treatment‐naïve samples allowed immunopathology study at the peak of disease without confounding effects of therapeutic heterogeneity. The comparison between aSLE and self‐matched follow‐up rSLE allowed immunophenotype monitoring along with disease progression and eliminated baseline immune variations among individuals. The single‐cell proteomic analysis of diverse immune phenotypes by CyTOF discovered that CD8^+^CD27^+^CXCR3^−^ T cell abundance was higher in rSLE than treatment‐naïve aSLE. Besides, the overactive effector function and autophagy behaviors of CD8^+^CD27^+^CXCR3^−^ T cells in rSLE were attenuated as compared with treatment‐naïve aSLE. Meanwhile, IL‐2 treatment reduces the expression of specific autophagy regulating proteins, especially in treatment‐naïve aSLE. In parallel, the effector function and autophagy behavior of CD8^+^CD27^+^CXCR3^−^ T cell is positively associated with clinical disease activity, including SLEDAI score.

CD27 expresses on most naïve and memory CD8^+^ T cells except for terminally differentiated effector T cells.^[^ ^17^ ^]^ Based on the CD27 expression and IFNγ secretion, these CD8^+^CD27^+^CXCR3^−^ T cells belong to a subtype of effector memory CD8^+^ T cells. Previous clinical studies have shown CD27 expression is beneficial for CD8^+^ T memory cell formation and long‐term survival,^[^ ^49^ ^]^ and persistent CD27 expression may indicate decreased effector function of CTLs.^[^ ^50^ ^]^ This might be the reason why CD8^+^CD27^+^CXCR3^−^ T cells in rSLE expressed higher CD27 but secreted lower IFNγ than treatment‐naïve aSLE. CD27 involves an immune response dependent on the expression of its ligand, CD70,^[^ ^51^ ^]^ which enables T cells to survive and develop into effector cells. Ligation of CD27 by CD70 in association with TCR signal would lead to down‐regulation of CD27.^[^ ^50^ ^]^ Besides, CD27 have been proved in promoting activated T cell survival in CD8^+^ T cells which might be beneficial to reduce immune response induced by cell debris from cell apoptotic necrosis.^[^ ^52^, ^53^ ^]^ The chemokine receptor CXCR3 is highly expressed on effector T cells and facilitates T cell recruitment and immune response.^[^ ^54^ ^]^ CXCR3 has been proved to be essential for efficient antiviral memory in lymph nodes, and CXCR3 deficiency could delay memory T cells’ recall responses.^[^ ^55^ ^]^ These results indicated the potential of the increased abundance of CD8^+^CD27^+^CXCR3^−^ T cell as a critical feature of SLE remission.

Both clinical trials and mechanistic studies have indicated that low‐dose IL‐2 can control autoimmune diseases and inflammation, including SLE.^[^ ^36^ ^]^ Based on previous research, IL‐2 affects the immune system, including driving T‐cell growth and inducing Treg differentiation via IL‐2 receptor, especially IL‐2Rα.^[^ ^56^ ^]^ Considering CD25 expressions are low in CD8^+^CD27^+^CXCR3^−^ T cells, effects of IL‐2 on this subset might be attributed to the expression of IL‐2Rβ (also known as CD122) on memory CD8^+^ T cells.^[^ ^57^ ^]^ In cells that lack expression of CD25, IL‐2 can associate with the dimeric IL‐2R directly (with a K_d_ of ≈10^−9^ M).^[^ ^58^ ^]^ Our demonstration of potent IL‐2 effects on CD8^+^CD27^+^CXCR3^−^ T cells adds a new dimension for IL‐2 associated immune regulation in SLE. Besides, the significant upregulation of Integrin αvβ3 in CD8^+^CD27^+^CXCR3^−^ T cells suggests that IL‐2 might affect this subset via integrin signal transduction.^[^ ^59^ ^]^ Integrin αvβ3 is a cell‐surface receptor and interacts with inflamed endothelium and stromal extracellular matrix (ECM) components like osteopontin. Integrin αvβ3 involves in TLR signaling pathway, exerting both proinflammatory and anti‐inflammatory functions.^[^ ^60^ ^]^ Therefore, the general upregulation of integrin αvβ3 in PBMCs needs to be analyzed in specific immune subsets, emphasizing the importance of single‐cell tools in monitoring therapeutic effects. For CD8^+^CD27^+^CXCR3^−^ T cells, the decreased effector function of PBMCs treated with IL‐2 corresponds to previous research that integrin β3 signaling could suppress IFNγ production via STAT1.^[^ ^61^ ^]^

Inhibition of autophagy biomarkers by IL‐2, especially in treatment‐naïve aSLE, might be due to activation of metabolic regulator mTOR.^[^ ^43^ ^]^ mTOR could inhibit autophagy activity by inhibiting autophagy‐initiating UNC‐5 like autophagy activating kinase (ULK).^[^ ^62^ ^]^ Autophagy activation is a mechanism for the survival of autoreactive B cells and is required for plasmablast.^[^ ^12^ ^]^ However, increased autophagy is also cytoprotective against antibody and interferon‐α induced podocyte injury.^[^ ^63^ ^]^ In terms of autophagy‐related therapeutic strategy in SLE, clinical research has proved the efficacy and safety of mTOR inhibitor sirolimus on active SLE treatment.^[^ ^64^ ^]^ Some studies also suggested that the inhibition of autophagy might regulate excessive proinflammatory macrophages in SLE.^[^ ^65^ ^]^

The exploratory approaches to evaluations of autophagy‐related biomarker expressions in PBMCs will help pave the way towards a systematic understanding of autophagy activity in SLE and unravel the complex relationship between autophagy and immune disorders in SLE pathogenesis. Considering the complex role of autophagy in SLE, the autophagy activity of CD8^+^CD27^+^CXCR3^−^ T cells might be a potential therapeutic target. Although IL‐2 could decrease the expression of autophagy‐related biomarkers, the short half‐life of IL‐2 in vivo and rapid uptake by CD25^+^ cells indicate IL‐2 is not an ideal treatment strategy for this potential target.

Identifying protein biomarkers of homeostasis in SLE patients is critical to predict the risk of recurrence or deterioration. Traditional analyses are able to distinguish the diseased patients from the healthy ones based on protein expression differences, but fail to detect the homeostasis of patient. Compared with conventional approach, DNB is able to explore the dynamical information of proteins and their correlations by utilizing high‐throughput time series datasets, based on the non‐linear dynamical theory. The basis for defining DNBs lies in the fact that biological networks are not static entities, but instead exhibit dynamic behavior in response to internal and external stimuli. This continuous‐time treatment caused nonlinear changes of protein expression in cells. In this study, we utilized DNB to quantify the dynamic response ability to IL‐2 stimulation. DNB proved not only to be compatible to judge the disease activity as static biomarkers but also to predict the remission and flare of SLE patients.

## 4 Conclusion

This study depicted the complex immune landscape and autophagy‐related molecules of the whole immune system in SLE. Our discoveries suggest the potential of CD8^+^CD27^+^CXCR3^−^ T cells as critical biomarkers of SLE remission and potential therapeutic target for SLE. Besides, we discovered IL‐2 treatment related DNB could predict the SLE remission and flare. Our findings may provide a new strategy in evaluating the efficacy of personalized treatment for SLE, which would be beneficial to innovative immunotherapy for SLE. Our findings could also provide new insights into understanding the complex mechanism of IL‐2 treatment to SLE patients.

## 5 Experimental Section

### Study Design and Sample Collection

8HCs were first recruited, 10 treatment‐naïve aSLE and 8 remission SLE (rSLE) patients from Shanghai Renji Hospital, Shanghai Jiao Tong University, in Shanghai, China (Tables S1,S2, Supporting Information). Peripheral blood mononuclear cells of these participants were measured via mass cytometry (CyTOF) for comprehensive immune phenotyping. Notably, the same 7 individuals were recruited at two different time points, one during active disease and one during remission (Table S2, Supporting Information). For dynamic monitoring of immune system variation, PBMCs of this cohort were treated with interleukin‐2 (IL‐2) (Table S2, Supporting Information) (Data shown in Figures 2, 3, 6).

For the cross‐validation of CyTOF results, 15 self‐matched active SLE (aSLE) and 24 weeks follow‐up samples, also 44 patients including aSLE, non‐paired rSLE, RA and AS were recruited to further investigate CD8^+^ T cell subsets from Shanghai Renji Hospital, Shanghai Jiao Tong University, in Shanghai, China (Table S5, Supporting Information). PBMCs of this cohort were analyzed via flow cytometry (Data shown in Figure 2; Figure S6, Supporting Information).

For further function investigation, PBMCs were recruited from another 2 aSLE and 2 rSLE and stimulated them with Leukocyte Activation Cocktail. These 4 samples were also measured via CyTOF (Table S2, Supporting Information) (Data shown in Figure 3).

For further function investigation, PBMCs from another 6 aSLE and 2 HCs were measured via scRNA‐seq (Data shown in Figure 4).

For further understanding of signaling pathway dysregulation, “Single‐cell RNA‐seq reveals cell type–specific molecular and Genetic Associations to Lupus” article data GSE174188_CLUES1_adjusted.h5ad.gz, data download address: https://www.ncbi.nlm.nih.gov/geo/download/?acc = GSE174188, from which 24 samples were randomly selected for data analysis in this paper was utilized.^[^ ^28^ ^]^ (Data shown in Figure 5).

For dynamic monitoring of immune system variation, PBMCs of the same cohort were treated with interleukin‐2 (IL‐2) (Data shown in Figures 7, 8).

All SLE patients mentioned above were diagnosed based on the criteria of the American College of Rheumatology.^[^ ^66^ ^]^ Based on the disease activity determined by the SLE Disease Activity Index (SLEDAI),^[^ ^67^ ^]^ these SLE patients were defined as aSLE (SLEDAI score > 5) and rSLE (SLEDAI score ≤ 5).

### PBMCs Isolation and In Vitro Cell Culture

PBMCs were isolated by density gradient centrifugation with Ficoll‐Paque PLUS Media (Thermo Fisher Scientific) within 4 h after collection (400 g, 20 min). Isolated PBMCs were washed with DMEM (Solarbio) (400 g, 5 min).

The PBMCs were separated into five tubes (2–3 million cells in each tube) marked from 1 to 5. Cells in tube 1 were stained with cisplatin (5 uM), fixed by PFA (1.6%) and diluted in freezing solution (cell staining buffer, CSB, 10% DMSO) under ‐80 °C for long‐term storage.

To monitor IL‐2 effects on PBMCs, cells in tubes 2–5 were stimulated with recombinant human IL‐2 via incubation in 1 ml PBMC media (DMEM, 5% FBS, 100 U ml^−1^ penicillin, and 100 U ml^−1^ streptomycin) containing IL2 (30 ng ml^−1^).^[^ ^13^ ^]^ Then the tubes were transferred into a 24‐well plate and incubated for 30 min, 1, 2, and 4 at 37 °C in 5% CO_2_. Last, cells were treated the same way as those in Tube 1.

For cytokine analysis, PBMCs were stimulated with 2ul Leukocyte Activation Cocktail, with BD GolgiPlug (BD) incubated in 1 ml PBMC media (DMEM, 10%FBS, with 100U ml^−1^ penicillin and 100U ml^−1^ streptomycin) for 4 h at 37 °C in 5% CO_2_ before cisplatin staining and PFA fixation. This cocktail contains the phorbol ester, PMA (Phorbol 12‐Myristate 13‐Acetate), a calcium ionophore (Ionomycin) and the protein transport inhibitor BD GolgiPlug (Brefeldin A) according to product description.

### Antibodies for This Study

Antibodies with pre‐labeled mass tags used for mass cytometry were purchased from Fluidigm. Other purified antibodies in carrier protein‐free PBS were purchased from Abcam, Arigo, Novus, or Biolegend (Table S3, Supporting Information). According to the manufacture's protocol, the metal tags were labeled using the MaxPAR antibody conjugation labeling kit (Fluidigm).

### Flow Cytometry Experiments

PBMCs were isolated by density gradient centrifugation with Ficoll‐Paque PLUS Media (Thermo Fisher Scientific). Then, PBMCs were incubated with Human TruStain FcX (Biolegend) for 10 min and antibodies (Table S3, Supporting Information) for a further 30 min successively. PBMCs were resuspended in PBS and analyzed by flow cytometry (BD Biosciences). Data were analyzed through FlowJo software (Treestar Inc.) and Cytobank.

### Sample Barcoding and Antibody Staining for CyTOF

To reduce signal variations produced during antibody staining and CyTOF analysis, two‐step barcoding strategies were employed. Before antibody staining, Cryopreserved PBMCs were thawed and stained with Human TruStain FcX (Biolegend) for 10 min to avoid non‐specific adsorption.

CD45 antibodies labeled with different metals tags were used to stain the five PBMC tubes of one person for 30 min. After washing with CSB twice, the PBMCs in the five tubes were pooled together. Blood samples of different people were barcoded using a Cell‐ID 20‐Plex Pd Barcoding Kit (Fluidigm) following the manufacturer's instructions. Then, all the blood samples were pooled together after palladium (Pd) barcoding.

The pooled cells were incubated with a surface antibody cocktail for 30 min. Before intracellular staining, eBioscience^TM^ Foxp3 / Transcription Factor Staining Buffer Set Kit (Thermo Fisher Scientific) was used for membrane permeabilization. Then, the PBMCs were incubated with an intracellular antibody cocktail for 30 min and washed twice using CSB.

The cells were diluted with 100ul PBS and incubated with 125 nM iridium‐ containing DNA intercalator (Fluidigm), then dissolved in Fix and Perm buffer overnight at 4 °C. This step aims to facilitate discrimination of single cells, debris, and dimers.

Then, the cells were washed twice with CSB and four times with distilled water, diluted with distilled water to ≈10^6^ cells ml^−1^, and filtered through a 70‐µm membrane. Before CyTOF analysis, EQ‐Beads were added at the ratio of 1:5 for data normalization.

### CyTOF Data Analysis

The cells were measured with CyTOF at an acquisition rate of ≈500 events s^−1^ and other data acquired parameters were set following instructions.^[^ ^68^ ^]^ According to the protocol, data acquisition, beads normalization, Pd debarcoding were performed with CyTOF Software (6.7, Fluidigm).^[^ ^41^ ^]^ Finally, separate 5 .fcs files were obtained for differently processed samples for each patient.

All .fcs files were uploaded to Cytobank (https://www.cytobank.org/, Cytobank, Inc., https://community.cytobank.org/cytobank/experiments/91618/gating). Living singlets were gated by selecting events with low 151,153 europium (Er), 195 cisplatin (Pt), and appropriate 191, 193 iridium (Ir) signal intensity. For anti‐CD45 debarcoding, 5 clusters with CD45 labeled with different metal tags were gated (Figure 4b). Then the .fcs files were split of each patient into 5 separate ones based on gated subpopulations. All separate .fcs files were downloaded from Cytobank for further analysis.

### ACCENSE Visualization for CyTOF

The Automatic Classification of Cellular Expression by Nonlinear Stochastic Embedding (ACCENSE) was used to cluster high‐dimensional CyTOF data.^[^ ^14^ ^]^ The PBMCs were automatically stratified into 43 different clusters depending on their expression levels of 21 surface biomarkers (Table S3, Supporting Information).

### scRNA‐seq Data Analysis

Single‐cell suspensions were loaded into microfluidic devices and scRNA‐seq libraries were constructed by GEXSCOPE Single‐Cell RNA Library Kit (Singleron Biotechnologies) per the manufacturing's instructions. Then individual libraries were diluted to 4 nM and pooled together for sequencing via an Illumina NovaSeq 6000 (illumina, US).

### scRNA‐seq Data Processing

The CellRanger (v6) was used to compare and quantify the original data, referring to the genome Human reference (GRCh38) dataset required for Cell Ranger (https://support.10xgenomics.com/single‐cell‐geneexpression/software/downloads/latest).

The data analysis including data normalization and cell clustering was implemented in an open source R toolkit Seurat (version 4.0).^[^ ^69^ ^]^ The DecantX was utilized to decontaminate ambient mRNA and filter out low quality cells (min.cell <3, min_feature < 3, percent.mt > 20, percent.mt < 0.1).^[^ ^70^ ^]^ The PBMCs were annotated into specific cell subtypes based on the reference dataset which provided the mRNA and surface protein information of PBMCs.^[^ ^69^ ^]^

For scRNA‐seq analysis of article data, single‐cell data from 24 samples were extracted and began to analyze, first converting the data into a Seurat object using the “anndata” package (version 0.7.5.6) in R (version 4.0.0), and then annotating 98,073 cells from 24 samples according to the annotation results in the original document, resulting in 48,456 T cells.

### scRNA‐seq Data: Sub‐Cell Type Analysis

Raw gene expression count of T cells/ PBMCs were extracted from previous cleaned data and create a new Seurat object. Gene expression matrices were normalized to total cellular read count, and highly variably genes (HVG) selected from the normalized data using Seurat SCTransform function with default parameters. Batch effects were observed and corrected using R package “harmony” (Version 1.0.0).^[^ ^71^ ^]^ Following dimensional reduction and clustering used same procedure for whole cell population.

### Identification of Marker Genes and Differential Expression Genes (DEG)

To identify marker genes for these cell types, the gene expression values of cells were compared from the cluster of interest to that of cells from the rest of clusters using the Seurat FindMarkers function with default parameter of “MAST”^[^ ^72^ ^]^ test. Marker genes were defined based on the following criteria: 1) the average expression value in the cluster of interest was at least 1.2‐fold higher than the average expression in the rest of clusters; 2) there were >10% of cells in the cluster of interest which were detectable; and 3) marker genes should have the highest mean expression in the cluster of interest compared to the rest of clusters.

To calculate DEG between three group of CD8^+^CD27^+^CXCR3^−^ cells, e.g., Seurat FindMarkers function with MAST method^[^ ^72^ ^]^ were applied for three group of cells with parameter ″min.pct = 0.01, logfc.threshold = 0.01″.

For marker genes and DEG lists, GO and pathway analyses were performed by R package ClusterProfile (V3.18.1)^[^ ^73^ ^]^ with parameter ″p_val_adj < 0.05, pvalueCutoff = 0.05, qvalueCutoff = 0.05″.

### Trajectory and RNA Velocity Analysis

Trajectory and pseudotime analysis were conducted by monocle3 (Version 1.0.0).^[^ ^74^ ^]^ The algorithms place the cells along a trajectory corresponding to a biological process by taking advantage of an individual cell's asynchronous progression under an unsupervised framework. The trajectories were visualized as UMAP plot.

### Gene Set Enrichment Analysis

Differentially expressed genes with adjusted pvalue < 0.05 were screened between different disease conditions of cell types via “FindMarkers” function. “clusterProfiler” was carried out to detect Gene Ontology (GO) and Kyoto Encyclopedia of Genes and Genomes (KEGG) pathways enriched by differentially expressed genes.^[^ ^75^ ^]^ The terms with false discovery rate (FDR) <0.05 were regarded as significant enrichment.

The unique genes and DEGs were analyzed via FindMarkers function in the Seurat package based on the MAST method.^[^ ^72^ ^]^ GSEA enrichment analysis (https://www.gsea‐msigdb.org/gsea/index.jsp) was performed using the clusterProfiler.^[^^73]^

### DNB Analysis

R package DNB was first used (version 0.1.0) to analysis molecular dynamical network of each sample, and then counted the frequency of the best dynamical protein markers for all samples, and found that 4 proteins in the top5 can robustly describe the homeostasis of healthy people. Consequently, the 4 proteins were used to calculate the DNB score of each patient at each time point.

### Statistical analysis

Normality test was first performed via Kolmogorov‐Smirnov tests. Then the p values of ACCENSE and manual gating data based on results of normality test was calculated. If the data obeyed the normality distribution, unpaired t tests between columns based on two‐tailed Welch's t‐tests, paired t tests between columns based on two‐tailed paired t tests and tests among columns based on Turkey's multiple comparison test was performed. On the contrary, if the data didn't obey the normality test, unpaired t tests between columns based on two‐tailed Mann‐Whitney test, paired t tests between columns based on two‐tailed Wilcoxon signed‐rank tests paired t tests, unpaired tests among columns based on Kruskal‐Wallis test, and paired t tests among columns based on Friedman tests were performed. Detailed values of data and analysis methods for each figure were provided in the source data (.xlsx).

## Conflict of interest

The authors declare no conflict of interest.

## Author Contributions

L.Z., F.D., and Q.J. contributed equally to this work. L.Z. performed the mass cytometry (CyTOF) experiments and analysis, and wrote the manuscript. F.D. performed the flow cytometry (FCM) experiments and analysis, and wrote the manuscript. Q.J. performed the DNB analysis, and wrote the manuscript. B.W. and Z.T. helped to write the MATLAB scripts for CyTOF analysist. X.M. and Y.Z. helped with sample recruitment and flow cytometry experiments. L.S., B.H., and W.S. contributed to experiment design and paper writing. Rui Song and Chunmei Wu contributed to supplemental experiments and followup reports. L.C., X.C., and X.D. contributed to the experiment design, supporting funds, and manuscript organization. X.D. was the principal corresponding author.

## Supporting information

Supporting Information

## Data Availability

The data that support the findings of this study are available in the supplementary material of this article.
