# Supplementary material for: Identification and Characterization of CD8+CD27+CXCR3− T Cell Dysregulation and Progression‐Associated Biomarkers in Systemic Lupus Erythematosus
Source: Adv Sci (Weinh). 2023 Oct 24;10(35):2300123. doi: 10.1002/advs.202300123 (PMC10724430; doi:10.1002/advs.202300123)
Supplement: Supplementary file 1 — Supporting Information [file ADVS-10-2300123-s001.pdf]

ADVANCED SCIENCE
Open Access

Supporting Information

for *Adv. Sci.*, DOI 10.1002/advs.202300123

Identification and Characterization of $CD8^{+}CD27^{+}CXCR3^{-}$
T Cell Dysregulation and Progression-Associated Biomarkers in Systemic Lupus Erythematosus

Lulu Zhang, Fang Du, Qiqi Jin, Li Sun, Boqian Wang, Ziyang Tan, Xinyu Meng, Baozhen Huang, Yifan Zhan, Wenqiong Su, Rui Song, Chunmei Wu, Luonan Chen, Xiaoxiang Chen* and Xianting Ding**

Identification and characterization of $CD8^{+}CD27^{+}CXCR3^{-}$ T cell dysregulation and progression-associated biomarkers in systemic lupus erythematosus

Supplementary Tables

Table S1. Demographics and clinical characteristics of HCs, aSLE and rSLE measured via CyTOF.

	HCs	aSLE	rSLE
Sex (M/F), n	M=1 , F=7	M=0 , F=10	M=0 , F=8
Age (years)		33.25±14.35	29.70±8.99
SLEDAI	-	9.33±2.81	2.80±1.03
WBC (10^9/L)	-	5.68±4.05	5.84±1.78
PLT (10^9/L)	-	190.10±88.91	241.78±78.06
ESR (mm/h)	-	48.50±26.22	11.89±5.86**
CRP (mg/L)	-	9.62±10.92	3.61±6.53*
C3 (g/L)	-	0.53±0.19	0.77±0.09*
C4 (g/L)	-	0.07±0.05	0.13±0.05*
dsDNA (IU/mL)	-	319.30±103.49	130.17±122.01
ANA (1:N)	-	830.00±494.66	391.11±367.57*
IgG (g/L)	-	18.93±5.09	12.72±2.38*
IgA (g/L)	-	3.67±1.75	1.70±1.29
IgM (g/L)	-	1.39±0.86	1.93±0.89
Medications, n (%)			
Hydroxychloroquine	-	-	8(100)
Prednisone	-	-	8(100)
Mycophenolate mofetil	-	-	1(12.5)
Cyclophosphamide	-	-	1(12.5)
Tacrolimus	-	-	1(12.5)

Unless stated otherwise data are means ± SD. Clinical parameters for patients with SLE were obtained by routine laboratory test. The p value is based on two-tailed Mann-Whitney test. *， $p<0.05$; **, $p<0.01$.

ANA,anti-nuclear antibodies; aSLE, active SLE; C3, complement 3; C4, complement 4; CRP, C-reactive protein; CyTOF, mass cytometry; ds-DNA, double-stranded DNA; ESR, erythrocyte sedimentation rate; F, female; HCs, healthy controls; M, male; PLT, platelets; rSLE, remission SLE; SD, standard deviation; SLEDAI, SLE disease activity index; WBC, white blood cells.

Table S2. Clinical characteristics of all SLE patients analyzed by CyTOF in this study.

No.	Disease activity	Sex	Age	SLE DAI	Mainifestations	WBC	PLT	ESR(	CRP(	C3	C4	dsDNA	ANA	IgG	IgA	IgM	Medications	Treated with IL2					PMA
						10^9/L		mm/h	mg/L)	(g/L)		(IU/mL)	(1:N)	(g/L)				un	0.5h	1h	2h	4h	
SLE-1	Active	F	14	8	Lupus enteritis, LN	5.9	275	105	14.7	0.613	0.063	446.81	1280	20.8	2.9	1.7	-	√	√	√	√	√	-
SLE-2	Active	F	34	8	Rash, fever, arthritis	6.09	331	36	1.3	0.848	0.131	376.36	1280	22.7	4.09	1.1	-	√	√	√	√	√	-
SLE-3	Active	F	33	10	LN	17	254	7	4.87	0.535	0.143	367.25	320	6.59	1.9	0.32	-	√	√	√	√	√	-
SLE-4	Active	F	37	14	LN	-	81	44	8.9	0.223	0.021	236.24	1280	19.1	2.55	1.26	-	√	√	√	√	√	-
SLE-5	Active	F	29	10	LN	2.86	-	43	7.8	0.334	0.017	385.7	1280	21.1	3.7	1.5	-	√	√	√	√	√	-
SLE-6	Active	F	36	12	NPSLE	3.94	210	59	40.8	0.764	0.076		1280	21.1	3.94	1.35	-	√	√	√	√	√	-
SLE-7	Active	F	15	13	LN	2.75	172	81	6.08	0.649	0.09	292.16	640	19.5	3.1	0.89	-	√	-	√	√	√	-
SLE-8	Active	F	14	9	Alopecia, rash,arthritis	3.11	114	46	-	0.459	0.023		640	13.6	1.62	0.65	-	√	√	√	√	√	-
SLE-9	Active	F	37	8	LN	4.76	258	36	7.89	0.54	0.127	117	320	22.9	3.3	1.66	-	√	√	√	√	√	-
SLE-10	Active	F	64	9	Rash, feve	4.28	73	66	2.3	0.32	0.02	332.95	1280	22	7.83	3.5	-	√	√	√	√	√	-
SLE-1	Remission	F	14	2	LN	7.8	168	12	3	0.726	0.17	-	320	13.8	4.01	2	Pred+HCQ+CTX	√	√	√	√	√	-
SLE-2	Remission	F	34	2		5.44	383	12	0.6	0.848	0.131	186	160	-	-	-	Pred+HCQ	√	√	√	√	√	-
SLE-3	Remission	F	33	2	LN	8.98	186	16	20.7	0.726	0.173	-	320	8.9	0.36	2	Pred+FK506+HCQ	√	√	√	√	√	-
SLE-4	Remission	F	40	4	Gastrointestinal vasculitis	3.4	161	23	3.1	0.746	0.085	73.45	320	-	-	-	Pred+MMF+HCQ	√	√	√	-	√	-
SLE-5	Remission	F	29	2	LN	7.11	251	7	0.5	0.69,	0.076	-	160	-	-	-	Pred+HCQ	√	√	√	√	√	-
SLE-6	Remission	F	36	4	LN	4.96	243	12	3.1	0.795	0.111	349.68	1280	15.9	0.91	2.97	Pred+HCQ	√	√	√	√	√	-
SLE-7	Remission	F	15	2	LN	5.3	225	2	1.1	0.649	0.07	-	640	11.9	1.84	1.1	Pred+HCQ	√	√	√	√	√	-
SLE-11	Remission	F	35	4	LN	4.27	207	14	0.2	0.83	0.16	107	160	13.8	1.02	2.79	Pred+MMF+HCQ	√	√	√	√	√	-
SLE-12	Active	F	42	6	Lymphadenitis	3.94	133	36	6.7	-	-	-	40	-	-	-	-	-	-	-	-	-	√
SLE-13	Active	F	44	9	LN	7.9	-	23	4.9	0.5	0.13	-	320	-	5.49	-	-	-	-	-	-	-	√
SLE-14	Remission	F	25	2	LN	5.34	352	9	0.2	0.94	0.22	44.2	160	12	2.08	0.74	Pred+HCQ	-	-	-	-	-	√
SLE-15	Remission	F	36	4	LN	-	-	-	-	-	-	20.7	-	-	-	-	Pred+HCQ	-	-	-	-	-	√

Clinical parameters for patients with SLE were obtained by routine laboratory test.

ANA, antinuclear antibodies; C3, complement 3; C4, complement 4; CRP, C-reactive protein; CTX, cyclophosphamide; CyTOF, cytometry by time-of-flight; dsDNA, double-stranded DNA; ESR, erythrocyte sedimentation rate; F, female; FK506, Tacrolimus; HCQ, hydroxychloroquine; IL-2, interleukin-2; LN, lupus nephritis; M, male; MMF, mycophenolate mofetil; NPSLE, neuropsychiatric systemic lupus erythematosus; PLT, platelets; Pred, prednisone; SLE, systemic lupus erythematosus; SLEDAI, SLE disease activity index; un, unstimulated; WBC, white blood cells.

Table S3. The list of antibodies used in this study.

Antigen	Clone	Manufacturer	Mass/FL	Description	Ratio	Application
CD45	HI30	Fluidigm	89	Barcoding for 4 h	1:100	CyTOF
GM-CSF	BVD2-21C11	Biolegend	141	Intracelluar Markers	1:50	CyTOF
CD19	HIB19	Fluidigm	142	Surface Markers	1:100	CyTOF
CD5	UCHT2	Fluidigm	143	Surface Markers	1:100	CyTOF
CD11b	ICRF44	Fluidigm	144	Surface Markers	1:100	CyTOF
CD45	HI30	Biolegend	145	Barcoding for 2 h	1:100	CyTOF
CD8	RPA-T8	Fluidigm	146	Surface Markers	1:100	CyTOF
CD11c	Bu15	Fluidigm	147	Surface Markers	1:100	CyTOF
CD1d	51	Biolegend	148	Surface Markers	1:100	CyTOF
ATG5		Novus	149	Intracelluar Markers	1:100	CyTOF
IL-4	FN50	Biolegend	150	Intracelluar Markers	1:50	CyTOF
CD123(IL-3R)	6H6	Fluidigm	151	Surface Markers	1:100	CyTOF
CD185	RF8B2	Fluidigm	153	Surface Markers	1:100	CyTOF
CD279 (PD-1)	EH12.2H7	Biolegend	154	Surface Markers	1:100	CyTOF
CD56	B159	Fluidigm	155	Surface Markers	1:100	CyTOF
CD183	G025H7	Fluidigm	156	Surface Markers	1:100	CyTOF
IFNγ	B27	Fluidigm	158	Intracelluar Markers	1:100	CyTOF
Foxp3	259D/C7	Fluidigm	159	Intracelluar Markers	1:50	CyTOF
CD69	FN50	Biolegend	160	Surface Markers	1:100	CyTOF
Ki67	Ki-67	Fluidigm	161	Intracelluar Markers	1:100	CyTOF
CD51/61	23C6	Biolegend	162	Surface Markers	1:100	CyTOF
CD45	HI30	Biolegend	163	Barcoding for 1 h	1:100	CyTOF
CD45	HI30	Biolegend	165	Barcoding for 0.5 h	1:100	CyTOF
LC3B	Polyclonal	Abcam	166	Intracelluar Markers	1:100	CyTOF
ATG7	Polyclonal	Arigo	167	Intracelluar Markers	1:50	CyTOF
RORγt	600214	Fluidigm	168	Intracelluar Markers	1:50	CyTOF
CD25(IL-2R)	2A3	Fluidigm	169	Surface Markers	1:100	CyTOF
CD3	SP34-2	Fluidigm	170	Surface Markers	1:100	CyTOF
CD45	HI30	Biolegend	171	Barcoding for 0h	1:100	CyTOF
IL-17A	BL168	Fluidigm	172	Intracelluar Markers	1:100	CyTOF
CD27	O323	Biolegend	173	Surface Markers	1:100	CyTOF
CD4	SK3	Fluidigm	174	Surface Markers	1:100	CyTOF
CD14	M5E2	Fluidigm	175	Surface Markers	1:100	CyTOF
CD196	G034E3	Fluidigm	176	Surface Markers	1:100	CyTOF
CD16	3G8	Fluidigm	209	Surface Markers	1:100	CyTOF
CD45	HI30	Biolegend	PE-Cy5	Surface Markers	1:100	FCM
CD3	SP34-2	Biolegend	FITC	Surface Markers	1:100	FCM
CD4	SK3	Biolegend	BV-590	Surface Markers	1:100	FCM
CD8	RPA-T8	BD	BV-421	Surface Markers	1:100	FCM
CD27	M-T271	BD	APC-H7	Surface Markers	1:100	FCM
CD16	Mouse IgG1, K	Biolegend	APC	Surface Markers	1:40	FCM

Note: CyTOF, mass cytometry; FCM, flow cytometry; FL: fluorescence; IL-2, interleukin 2; PBMCs: peripheral blood mononuclear cells; 0h samples, PBMCs without IL-2 treatment in vitro; 0.5 h samples, PBMCs treated with IL-2 for 0.5 h; 1 h samples, PBMCs treated with IL-2 for 1 h; 2 h samples, PBMCs treated with IL-2 for 2 h; 4 h samples, PBMCs treated with IL-2 for 4 h.

Table S4. Detailed immune phenotype for 43 clusters according to their lineage-related molecule expression patterns.

	Phenotype	CD19	CD5	CD11b	CD8a	CD11c	CD1d	CD123	CD185	CD279	CD56	CD183	FoxP3	RORC	CD25	CD3	CD27	CD4	CD14	CD196	CD16
Cluster 1	T follicular helper B cells	+	-	-	-	int	+	int	+	int	int	-	int	-	int	-	-	-	int	+	-
Cluster 2	$CD185^{+}CD183^{+}Foxp3^{+}$ B cells	+	-	-	-	int	+	int	+	int	int	+	+	-	int	-	-	-	int	+	-
Cluster 3	T follicular helper B cells	+	-	-	-	int	+	int	+	int	int	-	int	-	int	-	-	-	int	+	-
Cluster 4	/	+	+	+	+	+	+	+	+	+	+	+	+	+	+	+	+	+	+	+	+
Cluster 5	Follicular helper T cell precursor B cells	+	-	-	-	int	+	int	+	int	int	+	int	-	int	-	-	-	int	+	-
Cluster 6	pDCs	-	-	+	-	+	-	+	+	int	int	int	-	-	+	-	-	-	int	-	+
Cluster 7	Regulatory B cells	+	-	-	-	int	+	int	+	int	int	-	int	-	+	-	int	-	int	int	-
Cluster 8	pDCs	int	-	int	-	int	-	int	int	int	int	-	-	-	+	-	-	-	int	int	int
Cluster 9	$CD27^{+}CD183^{+}Foxp3^{+}$ B cells	+	-	-	-	int	-	int	int	int	int	+	+	int	int	-	+	int	int	+	int
Cluster 10	$CD11b^{+}CD123^{+}$ B cells	+	-	+	int	+	+	+	int	+	+	int	+	+	+	-	int	int	+	+	+
Cluster 11	$CD8^{+}$T cells	int	int	-	+	int	-	int	int	int	int	-	-	-	int	int	int	-	int	-	+
Cluster 12	$CD11b^{+}$ cells	int	int	+	+	+	+	int	int	+	int	int	int	+	int	-	int	int	int	int	+
Cluster 13	$CD11b^{+}CD123^{-}$ B cells	+	-	+	-	+	+	int	int	+	+	int	int	+	+	-	int	-	int	+	+
Cluster 14	$CD11b^{+}CD8^{+}$T cells	int	+	+	+	int	int	int	int	int	int	-	int	-	int	+	-	-	int	-	-
Cluster 15	$CD27^{+}CXCR3^{+}CD4^{+}$ T cells	-	+	-	-	int	-	int	int	int	int	+	int	int	+	+	+	+	int	int	-
Cluster 16	$CD27^{+}CXCR3^{-}CD8^{+}$T cells	int	+	int	+	int	int	int	int	int	int	-	int	int	int	+	+	-	int	int	-
Cluster 17	Follicular B	+	-	+	-	+	+	int	int	+	+	int	int	+	+	-	int	-	int	+	+
Cluster 18	$CD27^{-}CXCR3^{-}CD8^{+}$T cells	int	+	int	+	int	+	int	int	+	int	-	int	int	int	+	int	-	int	int	-
Cluster 19	$CD11b^{+}$ cells	int	-	+	int	+	+	int	int	+	int	int	-	+	int	-	-	-	int	int	+
Cluster 20	$CD4^{int}$ T cells	+	+	int	+	int	int	int	int	+	int	+	+	+	+	+	+	+	int	+	int
Cluster 21	$CXCR3^{int}$ $CD11b^{int}$ $CD8^{+}$T cells	int	int	int	+	int	int	int	int	int	int	int	int	-	int	+	-	-	int	-	-
Cluster 22	$CXCR3^{+}CCR6^{+}CD4^{+}$T cells	int	+	int	int	int	-	int	int	int	int	+	int	-	int	+	+	+	int	-	-
Cluster 23	$CD27^{+}CXCR3^{+}CD8^{+}$T cells	int	+	int	+	int	+	int	int	int	int	+	int	int	int	+	+	-	int	-	-
Cluster 24	$CD27^{-}CXCR3^{+}CD8^{+}$T cells	int	+	int	+	int	int	int	int	+	int	+	int	int	int	+	int	-	int	int	-
Cluster 25	$CD27^{+}CXCR3^{+}Foxp3^{+}CD8^{+}$T cells	int	+	int	+	int	+	int	int	int	int	+	+	+	int	+	+	int	int	+	-
Cluster 26	Regulatroy T cells	int	+	int	int	int	-	int	+	int	int	-	+	int	+	+	+	+	int	int	-
Cluster 27	mDCs	-	-	+	-	+	-	int	int	int	int	-	-	+	int	-	-	-	int	-	+
Cluster 28	mDCs	int	-	+	+	+	int	int	int	int	+	-	int	-	int	-	-	-	int	int	+
Cluster 29	Th17 cells	int	+	int	+	int	-	int	int	+	int	-	int	int	int	+	+	+	int	int	-
Cluster 30	$PD1^{+}CXCR3^{+}CD4^{+}$T cells	-	+	-	-	int	-	int	int	+	int	+	int	int	int	+	+	+	int	int	-
Cluster 31	mDCs	-	-	int	-	+	-	int	int	int	+	-	int	-	int	-	-	-	int	int	+
Cluster 32	Regulatroy T cells	-	+	-	-	int	-	int	int	int	int	-	+	int	+	+	+	+	int	int	-
Cluster 33	$PD1^{int}CD4^{+}$T cells	-	+	-	-	int	-	int	int	+	int	-	int	int	int	+	int	+	int	int	-
Cluster 34	$CD27^{+}CXCR3^{+}CD4^{+}$T cells	-	+	-	-	int	-	int	int	int	int	+	int	-	int	+	+	+	int	-	-
Cluster 35	$CD11b^{+}CD4^{+}$T cells	int	int	+	+	+	-	int	int	int	int	-	int	int	int	+	-	-	int	int	int
Cluster 36	Monocytes	int	-	+	-	+	+	+	int	int	int	-	int	int	int	-	-	int	+	int	int
Cluster 37	$CD27^{+}CXCR3^{-}CD4^{+}$T cells	-	+	-	-	-	-	int	int	int	int	-	int	int	int	+	+	+	int	int	-
Cluster 38	Monocytes	-	-	int	-	+	+	+	int	int	int	+	int	int	int	-	-	int	int	+	int
Cluster 39	Monocytes	int	-	+	int	+	+	+	int	+	+	+	int	int	int	-	-	+	+	int	int
Cluster 40	$CD4^{+}$T cells	-	int	int	-	int	-	int	int	int	int	int	int	int	int	+	int	-	int	-	int
Cluster 41	$CD27^{+}CXCR3^{int}CD4^{+}$T cells	-	+	-	-	int	-	int	int	int	int	int	int	int	int	+	+	-	int	int	-
Cluster 42	$CD4^{+}$T cells	-	int	int	-	int	-	int	int	int	int	-	int	int	int	+	-	-	int	-	int
Cluster 43	DNT	int	int	-	-	int	-	int	int	+	+	+	+	+	+	int	+	int	int	+	int

Table S5. 43 cell subpopulation frequency of individual PBMC samples across HCs, aSLEs, and rSLEs.

No.	Disease activity	Subpop. 1	Subpop. 2	Subpop. 3	Subpop. 4	Subpop. 5	Subpop. 6	Subpop. 7	Subpop. 8	Subpop. 9	Subpop. 10
HC-1	-	0.16	0.02	9.06	0	1.72	0.7	0.24	0.22	0.14	0.06
HC-2	-	0.02	0	1.88	0	2.64	0.84	2.28	0.1	0.32	0.48
HC-3	-	0.18	0.02	4.86	0.06	3.04	1.16	2.16	0.2	0.22	0.04
HC-4	-	0.12	0.02	2.62	0.96	3.24	1.56	0.74	0.1	0.22	1.18
HC-5	-	0.08	0.02	4.28	0.04	2.2	2.52	0.66	0.08	0.12	2.1
HC-6	-	0.06	0.02	1.7	0.02	2.14	1.96	1.7	0.16	0.2	7.62
HC-7	-	0.04	0.06	3.12	0.2	3.12	1.44	0.7	0.06	0.22	3.1
HC-8	-	0.08	0	5.54	0.2	3.54	1.72	0.78	0.12	0.22	6.84
SLE-1	Active	0.04	0	0.9	0.02	0.94	0.6	3.46	0.1	5.82	0.36
SLE-2	Active	0.06	0.04	2.32	0.02	2.66	0.28	0.5	0.38	2.14	0.1
SLE-3	Active	0	0	0.52	0	0.2	1.26	0.62	0.3	0.04	2.2
SLE-4	Active	0	0	3.06	0	0.92	0.28	0.62	0.1	4.16	0
SLE-5	Active	0.02	0.02	1.22	0.02	8.64	1.08	0.16	0.2	1.44	0.44
SLE-6	Active	0.06	0.06	5.5	0	6.84	0.58	0.86	0.12	2.64	0
SLE-7	Active	0.06	0	3.52	0.96	2.4	0.26	1.82	0.06	0.74	0.06
SLE-8	Active	0.04	0	2.84	0	1.02	0.36	0.92	0.16	1.8	0.02
SLE-9	Active	0.2	0.12	9.26	0	5.44	0.16	0.5	0.02	1.36	3.98
SLE-10	Active	0	0	0.04	0.02	0.02	0.04	0.42	0	0.08	1.56
SLE-1	Remission	0.02	0	0.9	0	1.02	0.6	0.32	0.08	0.14	0.08
SLE-2	Remission	0.06	0.04	2.44	0	3.72	0.08	0.12	0.36	0.6	0
SLE-3	Remission	0.08	0	2.72	0	0.58	1.1	0.44	0.22	0.12	0.26
SLE-4	Remission	0	0	0.02	0.18	0	0.54	1.2	0.1	0.26	1.14
SLE-5	Remission	0.16	0	3.74	0	4.3	1.2	0.52	0.06	1.02	4.44
SLE-6	Remission	0.06	0.06	3.28	0.02	4.92	1.42	0.92	0.18	0.32	0.34
SLE-7	Remission	0.14	0.04	9.8	0	5.26	0.34	1.12	0.06	0.1	0
SLE-11	Remission	0.12	0	1.88	0.04	0.06	0.94	6.82	0.18	0.16	0
Passed normality		No	No	No	No	Yes	Yes	No	Yes	No	No
One-way ANOVA methods		Kruskal-Wallis test	Kruskal-Wallis test	Kruskal-Wallis test	Kruskal-Wallis test	Turkey's multiple comparison test	Turkey's multiple comparison test	Kruskal-Wallis test	Turkey's multiple comparison test	Kruskal-Wallis test	Kruskal-Wallis test
HC vs aSLE		0.1654	>0.9999	0.6479	0.2893	0.981	0.0007	>0.9999	0.9529	0.0806	0.3923
HC vs rSLE		>0.9999	>0.9999	0.9091	0.2144	0.9795	0.0200	>0.9999	0.8712	>0.9999	0.1778
aSLE vs rSLE		0.4577	>0.9999	>0.9999	>0.9999	0.9196	0.4374	>0.9999	0.9706	0.1345	>0.9999

No.	Disease activity	Subpop. 11	Subpop. 12	Subpop. 13	Subpop. 14	Subpop. 15	Subpop. 16	Subpop. 17	Subpop. 18	Subpop. 19	Subpop. 20	Subpop. 21
HC-1	-	0.16	0.02	0.28	1.06	0.24	0.58	0.18	0.48	1.02	0.88	0.36
HC-2	-	0.42	0.16	0.98	1.02	0.4	0.44	0.4	0.44	1.68	0.32	0.54
HC-3	-	0.3	1.14	0.2	0.58	0.2	0.7	0.06	0.6	1.68	0.08	0.72
HC-4	-	0.24	4.18	1.76	0.22	0.42	0.4	0.34	0.46	6.22	0.02	0.58
HC-5	-	0.1	0.06	0.94	0.14	0.06	1.5	0.36	1.46	0.5	2.64	0.38
HC-6	-	0.1	0.22	4.26	0.06	0.02	0.7	1.34	0.28	2.44	0.28	0.04
HC-7	-	0.34	0.12	1.98	0.22	0.14	3.3	0.12	1.3	1.26	0.3	0.64
HC-8	-	0.14	0.12	1.66	0.12	0.06	1.76	0.08	0.92	0.7	0.28	0.22
SLE-1	Active	0.26	4.54	0.88	0.8	0.12	0.98	0.2	0.74	6.78	0.32	0.54
SLE-2	Active	0.5	0.06	0.22	0.34	0.12	1.26	0.08	1.56	1.16	1.7	0.46
SLE-3	Active	0.32	0.32	4.36	1.72	0.06	0.92	0.92	1.14	15.4	0.68	1
SLE-4	Active	0.48	0.74	0.02	0.14	0.02	0.38	0	0.6	1.24	0.1	0.66
SLE-5	Active	0.38	0.04	0.4	0.12	0.02	0.52	0.08	1.16	0.2	0.44	1.3
SLE-6	Active	0.32	0.38	0.1	0.46	0.22	1.12	0.04	1.24	0.82	0.3	0.96
SLE-7	Active	0.38	2.3	0.44	1.98	0.08	1.98	0.46	1.92	8.46	0.06	1.34
SLE-8	Active	0.64	0.54	0.02	1.12	0.06	2.62	0.24	3.12	0.74	0.34	1.96
SLE-9	Active	0.14	0.26	3.5	0.42	0	2.26	0.56	1.06	2.44	0.32	0.18
SLE-10	Active	0.2	10.2	2.9	3.16	0.04	1.94	0.18	1.24	14	0	0.38
SLE-1	Remission	0.64	1.02	0.24	5.26	0.1	2.7	0.06	1.82	1.22	0.04	1.06
SLE-2	Remission	0.54	0.02	0.12	0.02	0.08	0.5	0.04	0.34	0.46	1.18	0.26
SLE-3	Remission	0.46	0.18	0.8	2.94	0.02	3.88	0.08	1.86	3.2	1.8	1.2
SLE-4	Remission	0.2	8.98	2.18	1.78	0.06	2.16	0.22	1.24	10.48	0.12	0.76
SLE-5	Remission	0.26	0.06	2.04	0.2	0.1	2.36	0.26	1.58	0.72	0.54	0.5
SLE-6	Remission	0.16	2.24	0.9	0.54	0.2	1.5	0.2	0.28	5.12	0.06	0.36
SLE-7	Remission	0.56	0.86	0.04	3.48	0.14	3.36	0.08	1.6	0.78	0.16	1
SLE-11	Remission	0.32	0.28	0.04	3.86	0.02	6.8	0	1.16	1.14	0.06	0.32
Passed normality		Yes	No	No	No	Yes	No	No	No	No	No	Yes
One-way ANOVA methods		Turkey's multiple comparison test	Kruskal-Wallis test	Kruskal-Wallis test	Kruskal-Wallis test	Turkey's multiple comparison test	Kruskal-Wallis test	Kruskal-Wallis test	Kruskal-Wallis test	Kruskal-Wallis test	Kruskal-Wallis test	Turkey's multiple comparison test
HC vs aSLE		0.1604	0.5459	0.8467	0.6671	0.05	>0.9999	>0.9999	0.1904	>0.9999	>0.9999	0.0849
HC vs rSLE		0.0911	>0.9999	0.6067	0.1181	0.1217	0.0426	0.3232	0.2485	>0.9999	>0.9999	0.4714
aSLE vs rSLE		0.9062	>0.9999	>0.9999	>0.9999	0.9391	0.1647	0.7349	>0.9999	>0.9999	>0.9999	0.5904

No.	Disease activity	Subpop. 22	Subpop. 23	Subpop. 24	Subpop. 25	Subpop. 26	Subpop. 27	Subpop. 28	Subpop. 29	Subpop. 30	Subpop. 31	Subpop. 32
HC-1	-	4.88	1.18	0.92	1.4	0.8	0.54	1.68	0.36	1.08	2.14	0.84
HC-2	-	3.86	1.3	1.06	0.76	0.32	0.44	5.04	0.2	1.5	1.64	0.46
HC-3	-	6.1	2.26	2.1	0.88	0.58	0.96	0.72	0.62	0.94	1.1	0.16
HC-4	-	5.16	1.96	1.44	0.56	0.44	2.24	2.3	0.4	0.74	0.98	0.16
HC-5	-	1.06	1.6	0.68	3.68	1.46	0.06	0.98	0.22	1.44	2.08	0.7
HC-6	-	0.66	0.56	0.06	1.16	0.46	0.58	0.54	0.06	0.62	6.6	0.7
HC-7	-	1	2.48	0.9	5.54	1.08	0.14	0.38	0.14	0.96	1.96	0.8
HC-8	-	0.92	1.28	0.34	1.96	1.36	0.12	1.3	0.2	0.82	4.04	1.16
SLE-1	Active	2.64	2.54	1.38	0.92	0.64	5.72	1.08	0.32	1.48	1.02	0.5
SLE-2	Active	1.96	1.36	0.8	4.96	0.54	0.44	0.2	0.22	1.54	2.32	0.44
SLE-3	Active	1.66	1.88	1.98	0.54	0.14	3.74	1.12	0.04	0.96	1.78	0.32
SLE-4	Active	2.54	0.22	0.4	0.18	0.2	28.22	0.1	0.26	0.82	0.54	0.18
SLE-5	Active	0.7	1.4	2.94	5.12	0.26	0.04	0.34	0.1	4.16	2.14	0.28
SLE-6	Active	4.18	1.54	1.68	1.6	0.74	1.08	0.4	0.68	2.02	0.66	0.28
SLE-7	Active	2.12	2.34	1.94	0.76	0.76	2.88	0.18	0.7	1.78	0.84	0.22
SLE-8	Active	1.22	1.3	2.06	1.12	1.66	0.68	0.58	1.04	1.64	0.82	0.6
SLE-9	Active	0.26	0.86	0.26	1.6	0.36	0.86	0.12	0.14	0.56	1.68	0.34
SLE-10	Active	0.64	0.3	0.34	0.06	0.96	6.72	0.12	1.1	0.92	0.06	0.34
SLE-1	Remission	2.22	2.36	1.02	0.58	1.7	0.32	1.32	0.74	0.9	0.48	0.46
SLE-2	Remission	5.06	0.2	0.32	0.6	0.64	0.5	0.44	0.3	0.46	3.74	0.86
SLE-3	Remission	1	1.48	1.12	1.48	1.58	1.02	2.34	0.64	0.6	2.42	0.58
SLE-4	Remission	2.62	1.66	0.96	0.24	1.14	2.36	0.48	0.96	0.78	0.24	0.42
SLE-5	Remission	0.94	1.3	0.7	3.02	1.36	0.12	0.48	0.46	2.16	2.28	0.54
SLE-6	Remission	3.16	3.08	1.1	0.74	1.14	1.22	2.02	0.6	1.06	1.18	0.18
SLE-7	Remission	2.08	3.34	1.32	1.68	1.1	0.42	0.78	0.88	1.06	1.16	0.46
SLE-11	Remission	0.98	0.18	0.24	0.04	2.62	1.96	1.98	2.24	0.38	0.84	1.16
Passed normality		No	Yes	Yes	No	Yes	No	Yes	No	Yes	No	Yes
One-way ANOVA methods		Kruskal-Wallis test	Turkey's multiple comparison test	Turkey's multiple comparison test	Kruskal-Wallis test	Turkey's multiple comparison test	Kruskal-Wallis test	Turkey's multiple comparison test	Kruskal-Wallis test	Turkey's multiple comparison test	Kruskal-Wallis test	Turkey's multiple comparison test
HC vs aSLE		0.968	0.8786	0.3924	>0.9999	0.709	0.0995	0.0428	0.9227	0.2407	0.1782	0.0983
HC vs rSLE		>0.9999	0.9585	0.964	0.5244	0.0595	>0.9999	0.7104	0.0267	0.9688	0.7988	0.9511
aSLE vs rSLE		>0.9999	0.7196	0.2641	>0.9999	0.0076	0.5605	0.2119	0.247	0.1569	>0.9999	0.1761

No.	Disease activity	Subpop. 33	Subpop. 34	Subpop. 35	Subpop. 36	Subpop. 37	Subpop. 38	Subpop. 39	Subpop. 40	Subpop. 41	Subpop. 42	Subpop. 43
HC-1	-	0.46	13.36	0.88	3.18	2.08	1.24	0.94	0.74	0.84	0	0.06
HC-2	-	0.36	6.42	3.84	2.96	0.56	1.12	0.9	2.14	1.4	0.04	0.04
HC-3	-	0.4	4.74	1.78	0.78	0.68	1.16	1.48	1.8	1	0	0
HC-4	-	0.42	5.2	0.56	0.52	0.74	1.14	1.44	0.44	0.58	0.02	0.02
HC-5	-	0.74	1.9	0.4	0.78	1.18	0.42	0.28	0.1	0.88	0	0.38
HC-6	-	0.46	1.82	0.04	1.5	1.16	1.82	0.94	0.1	0.28	0	0.12
HC-7	-	0.84	2.92	0.2	0.62	2.9	0.68	1.1	0.22	0.96	0	0.08
HC-8	-	0.5	2.04	0.16	1.3	2.3	1.36	0.58	0.14	0.66	0.04	0.06
SLE-1	Active	0.8	2.64	0.5	1.46	0.86	0.18	0.32	0.8	0.68	0.02	0
SLE-2	Active	0.66	5.94	0.38	3.86	1.08	0.5	2.86	0.5	1.04	0.02	0.24
SLE-3	Active	0.26	1.74	0.32	0.3	0.36	0.2	0.12	0.3	0.32	0	0.04
SLE-4	Active	0.56	4.94	0.66	0.14	1.52	0.18	0.12	0.54	0.38	0.02	0
SLE-5	Active	0.8	2.28	0.26	0.62	0.68	0.3	1.28	0.34	1.16	0	0.48
SLE-6	Active	0.94	7.74	0.58	0.3	1.88	0.44	0.38	0.64	0.68	0	0.1
SLE-7	Active	1.66	2.66	0.18	1.26	0.94	0.34	0.8	0.12	0.56	0	0.02
SLE-8	Active	2.02	2.12	0.38	1.42	4.54	1.22	2.2	0.38	0.62	0.02	0.06
SLE-9	Active	1.2	0.58	0.86	0.4	1.08	0.2	1.74	0.92	0.78	0.04	0.06
SLE-10	Active	2.18	0.28	0.28	0.7	0.94	0.04	0.24	0.16	0.24	0.02	0
SLE-1	Remission	0.84	1.26	0.94	4.82	1.1	0.64	6.22	1.02	1.1	0.02	0.02
SLE-2	Remission	0.38	6.68	0.94	0.1	1.82	0.22	0.06	0.1	0.36	0	0.02
SLE-3	Remission	1.18	0.92	0.38	6.14	2.22	0.6	1.08	0.3	0.58	0	0.08
SLE-4	Remission	0.68	1.9	0.4	2.1	1.7	0.36	0.9	0.18	0.26	0	0.02
SLE-5	Remission	1.54	1.68	0.04	2.58	1.92	1.02	3.8	0.2	1.18	0	0.3
SLE-6	Remission	0.48	4.48	0.64	1.78	1.7	0.64	1.98	1.02	2.04	0.02	0
SLE-7	Remission	1.5	2.76	0.18	0.84	1.7	0.42	1.18	0.12	0.24	0	0.02
SLE-11	Remission	2.92	0.56	0.4	4.26	9.28	0.36	0.12	0.3	0.96	0	0.02
Passed normality		No	No	Yes	Yes	No	Yes	Yes	No	Yes	No	No
One-way ANOVA methods		Kruskal-Wallis test	Kruskal-Wallis test	Turkey's multiple comparison test	Turkey's multiple comparison test	Kruskal-Wallis test	Turkey's multiple comparison test	Turkey's multiple comparison test	Kruskal-Wallis test	Turkey's multiple comparison test	Kruskal-Wallis test	Kruskal-Wallis test
HC vs aSLE		0.0669	0.9579	0.2907	0.8256	>0.9999	0.0003	0.9967	>0.9999	0.6554	>0.9999	>0.9999
HC vs rSLE		0.1006	0.3064	0.3946	0.1655	0.5233	0.0062	0.336	>0.9999	0.9973	>0.9999	0.7986
aSLE vs rSLE		>0.9999	>0.9999	0.9889	0.0427	0.0893	0.5434	0.3354	>0.9999	0.6098	0.4588	>0.9999

Methods for normality test: Kolomogorov-Smirnov test

Table S6. Clinical characteristics of paired SLE patients analyzed by flow cytometry.

ID	Date	CRP	ESR	C3/C4	dsDNA	24-hour urinary protein	Random urinary protein	WBC	platelet
		(mg/L)	(mm/h)		(IU/ml)	g/24 hours		10^9/L	
1	2021/4/14	<2.5	11	0.047		4.372	++++	13.25	395
2	2021/3/15	0.2	3	1.05/0.25	14.4	5.216	++++	17.57	232
3	2021/3/29	1	17	0.604/0.173	16.46	2.71	++	9.57	274
4	2021/3/29	0.5	16	0.662/0.163	15.01	0.761	++	3.92	190
5	2021/3/29	6.7	29	1.02/0.219	6.19	7.394	+++	13.18	269
6	2021/3/22	3.1	45	0.482/0.03	>100	0.866	+	2.22	171
7	2021/3/29	3.5	57	1.4/0.326	15.14	16.68	+++	8.19	237
8	2021/3/15	1.5	19	0.319/0.077	>100	3.468	+++	8.16	178
9	2021/3/29	1	31	0.401/0.039	>100	0.163	neg	5.85	301
10	2021/3/22	1.4	10	0.817/0.149	>100	0.042	+-	6.42	191
11	2021/3/22	1.8	18	0.987/0.26	22.11	0.255	neg	8.76	271
12	2021/3/29	0.6	6	0.864/0.081	31.61	1.098	++	13.38	244
13	2021/3/22	0	7	0.735/0.1	25.54	0.142	neg	4.91	317
14	2021/3/29	2.6	19	1.19	14.13	1.587	+	16.41	327
15	2021/3/15	0.2	7	0.832/0.124	17.01	0.135	+-	8.69	202
1	2021/8/9	5.2	30	0.994/0.059	160.9	1.595	++	10.2	321
2	2021/8/16	0.7	3	0.953/0.289	14.33	1.861	++	15.55	213
3	2021/10/11	0.4	19				+	6.24	9.6
4	2021/10/11	0.5	14	0.661/0.133	18.46	0.204	++	3.92	168
5	2021/10/11					0.915			
6	2021/10/18	2.5	14	0.743/0.121	15.92	0.059	+-	5.58	180
7	2021/10/25	1.7	16			0.229	neg	6.5	257
8	2021/10/25	2.4	44	0.395/0.094	79.44	1.276	+++	10.07	166
9	2021/10/25	2.6	25	0.51/0.073	>100	1.779	+++	6.36	271
10	2021/11/1	2.2	15	0.769/0.153	60.42	0.02046	neg	7.41	211
11	2021/11/8	10.1	45	1/0.301	48.59	0.038	+-	7.43	309
12	2021/11/15	0.2	4	0.735/0.089	35.66	0.096	+	8.5	275
13	2021/11/29	0.2	11	0.788/0.136	31.99	0.131	+-	5.85	284
14	2022/1/24	1.6	14	1.14/0.349	18.94	0.145	neg	9.23	340
15	2022/1/17	1.4	8	0.9/0.087	14.27	0.102	+-	7.29	195

Supplementary Figures

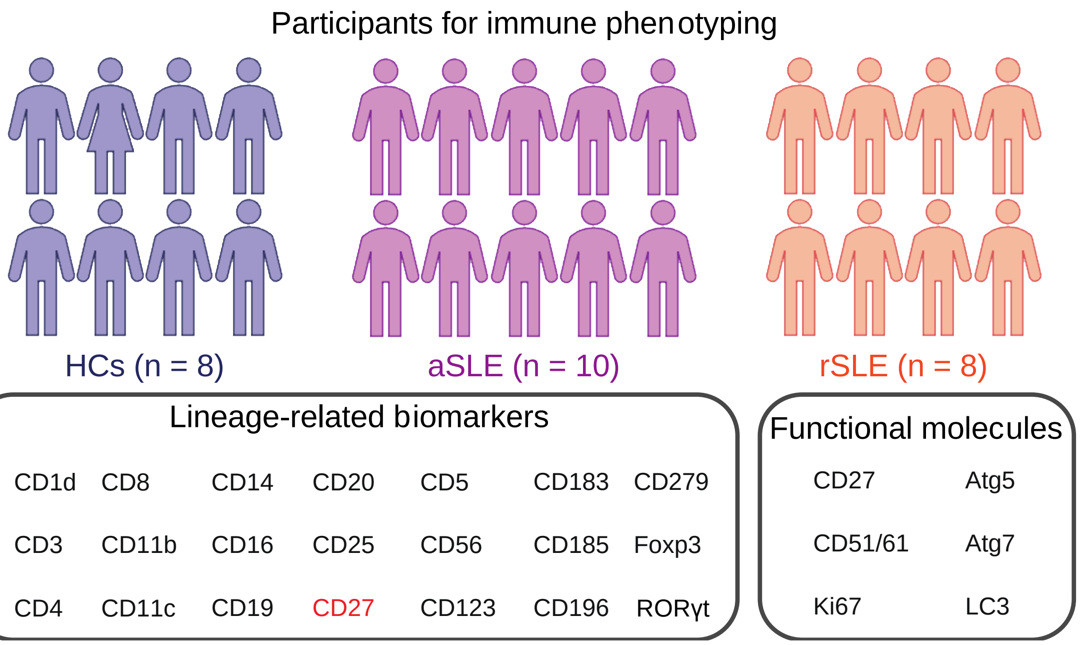

Figure S1. Recruited SLE patients are recruited and analyzed molecules via CyTOF for immune cell profiling at the single-cell level. aSLE, active SLE patients; HCs, healthy controls; rSLE, remission SLE patients; CyTOF, mass cytometry.

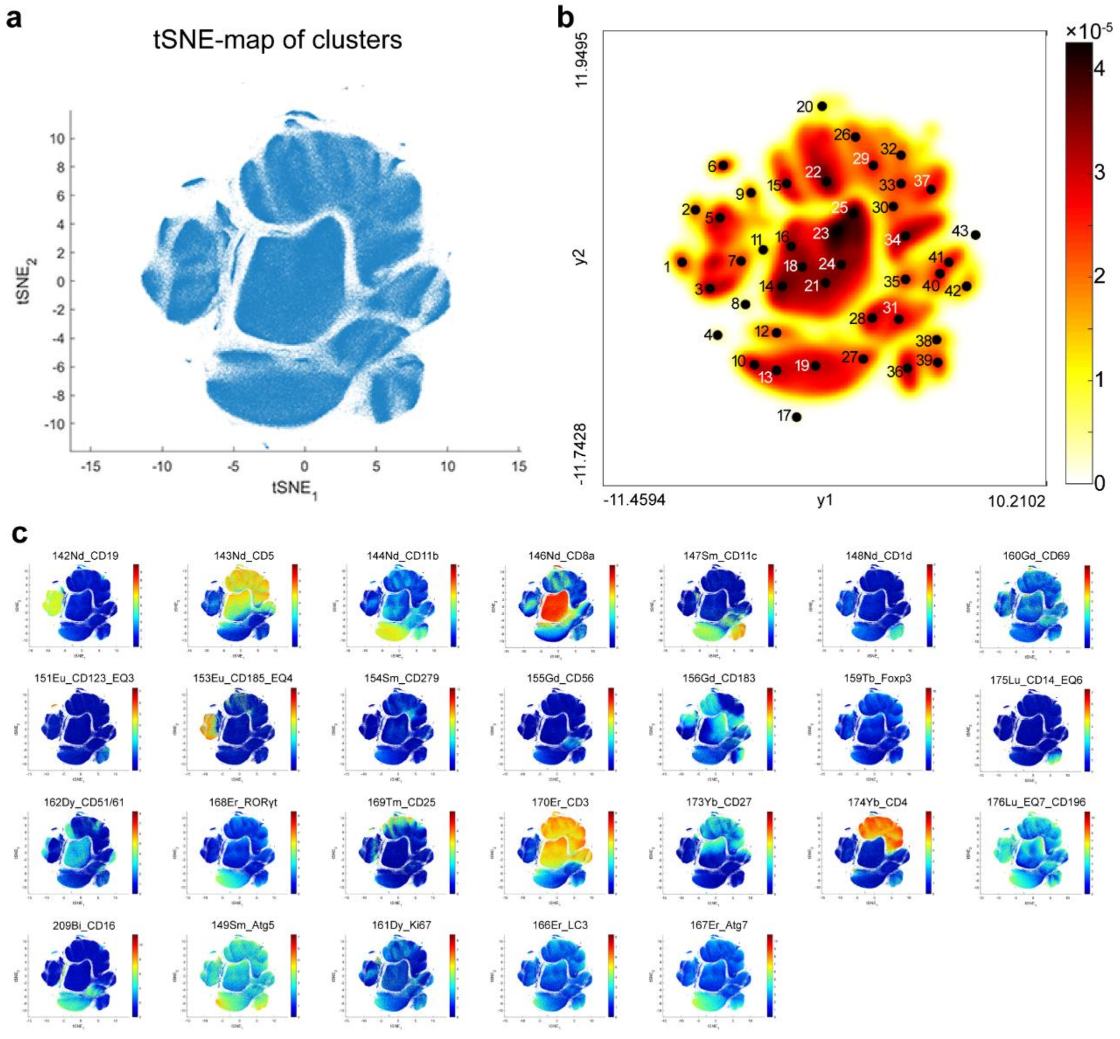

Figure S2. ACCENSE automatically partitions the PBMCs into 43 clusters. The overview (A) and the number-annotated t-SNE map (B) are shown. C, Single-cell t-SNE profiling of indicated molecules. ACCENSE, Automatic Classification of Cellular Expression by Nonlinear Stochastic Embedding; PBMCs, peripheral blood mononuclear cells; t-SNE, t-distributed stochastic neighbor embedding.

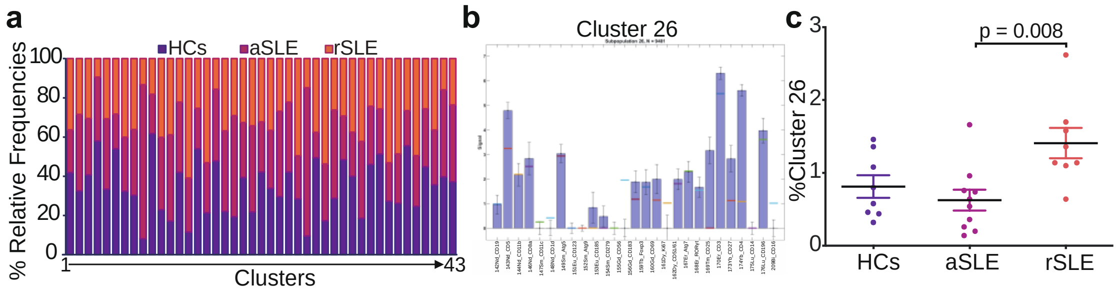

Figure S3. Characterization of Cluster 26 (CD4^{+}CD25^{+}Foxp3^{-}) Treg cell cluster. (A) Relative cell frequencies of 43 clusters across HCs, treatment-naïve aSLE and rSLE. (B) Combined molecule expression patterns of Clusters 26. (C) Individual cell abundance of Clusters 26. aSLE, active SLE patients; HCs, healthy controls; rSLE, remission SLE patients; SLE, systemic lupus erythematosus; Treg, regulatory T.

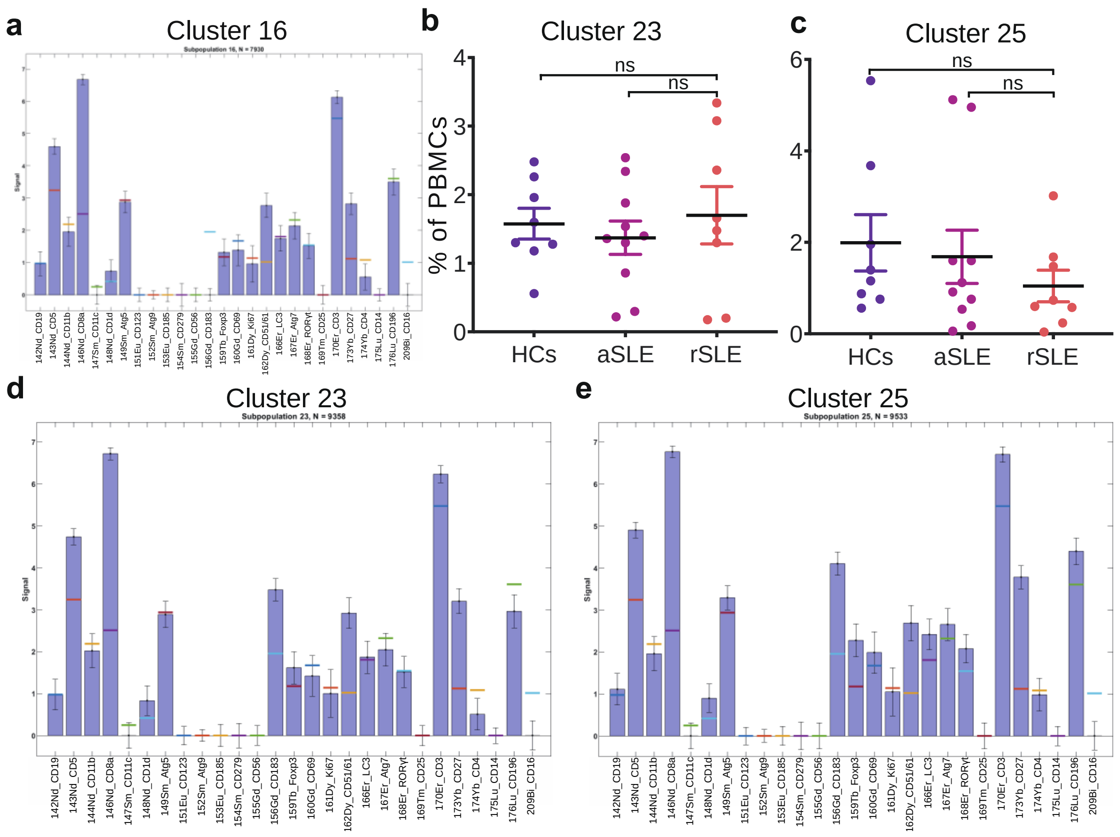

Figure S4. Characterization of CD8⁺CD27⁺ T cells. (A) Individual cell frequency of Clusters 16 across 3 groups. (B) Combined molecule expression patterns of Clusters 16. The manual gating strategy of Cluster 16 ($CD8^{+}CD27^{+}CXCR3^{-}$ T) cells. (C, D) Individual cell abundance of Clusters (C) 23 and (D) 25 across 3 groups. In the box plot, the data are presented as Mean±SEM and the p value is based on Tukey's multiple comparisons test, post-test $p < 0.05$. (E, F) Combined molecule expression patterns of Clusters (E) 23 and (F) 25. aSLE, active SLE patients; HCs, healthy controls; rSLE, remission SLE patients; SLE, systemic lupus erythematosus.

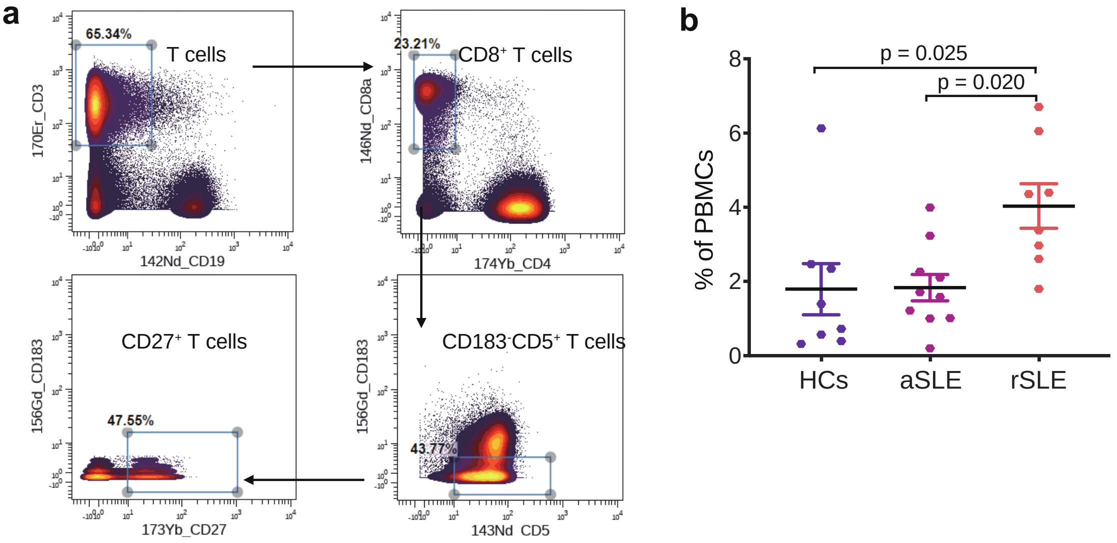

Figure S5. Characterization of Cluster 16 (CD8$^+$CD27$^+$CXCR3$^-$ T) cells. (A) The manual gating strategy of Cluster 16 (CD8$^+$CD27$^+$CXCR3$^-$ T) cells. (B) Manually gated Cluster 16 (CD8$^+$CD27$^+$CXCR3$^-$ T) cells in PBMCs across 3 groups. In the box plot, the data are presented as Mean±SEM and the p value is based on Tukey's multiple comparisons test, post-test $p < 0.05$.

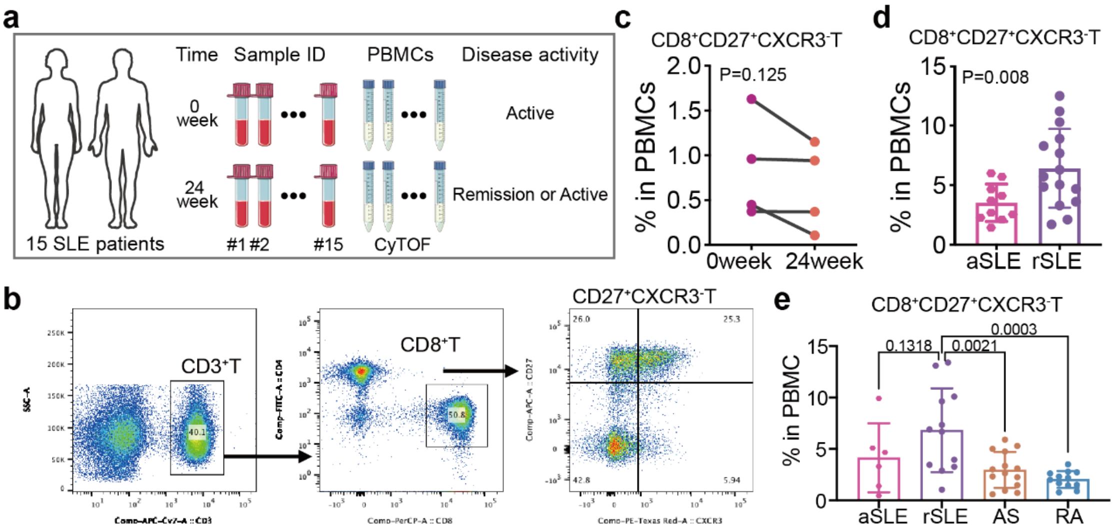

Figure S6. Flow cytometry of another 15 SLE patients further verified the elevated frequency of Cluster 16 (CD8$^+$CD27$^+$CXCR3$^-$ T) cells. (A) PBMCs of another 15 SLE patients were recruited. (B) Manual gating strategy of CD8$^+$CD27$^+$CXCR3$^-$ T cells.

(C) 4 aSLE in progression after 6-month follow-up showed decreased frequency of $CD8^{+}CD27^{+}CXCR3^{-}$ T cells. For the paired sample t-test, p value is based on two-tailed Wilcoxon signed-rank tests. (D) Individual CD8+CD27+CXCR3- T cell abundance among total PBMCs across unpaired aSLE (n=10) and rSLE (n=15). In the box plot, the data are presented as Mean±SEM and the p value is based on Welch's test. (E) Individual CD8+CD27+CXCR3- T cell abundance among total PBMCs across aSLE (n=6), rSLE (n=12), AS (n=14) and RA(n=12). In the box plot, the data are presented as Mean±SEM and the p value is based on Sidak’s post-hoc test. AS, ankylosing spondylitis; aSLE, active SLE patients; PBMCs, peripheral blood mononuclear cells; RA, rheumatoid arthritis; rSLE, remission SLE patients; SLE, systemic lupus erythematosus.

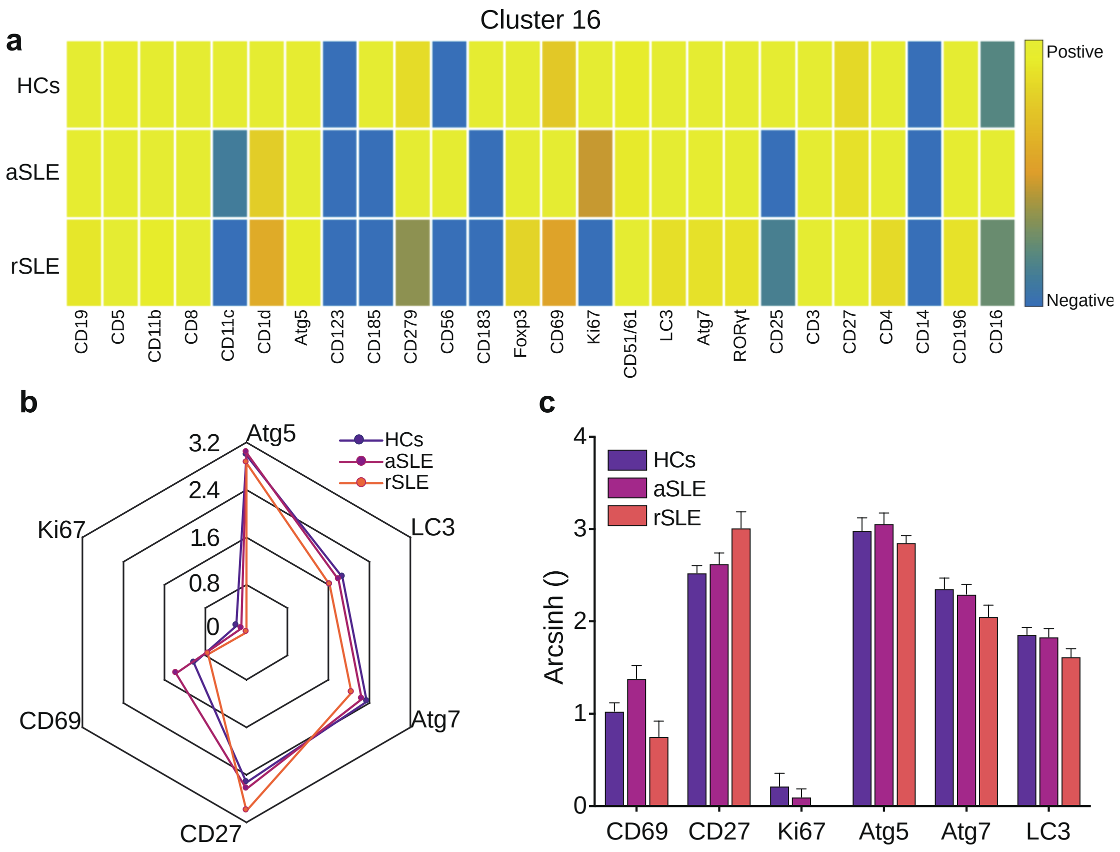

Figure S7. Cluster 16 ($CD8^{+}CD27^{+}CXCR3^{-}$ T) cells display enhanced effector function. (A) Relative expressions of all biomarkers of $CD8^{+}CD27^{+}CXCR3^{-}$ T cells across 3 groups. (B) Expressions of functional biomarkers of Cluster 16 cells with 0.8 intervals. (C) 6 functional signatures in 3 groups are presented as Mean±SEM.

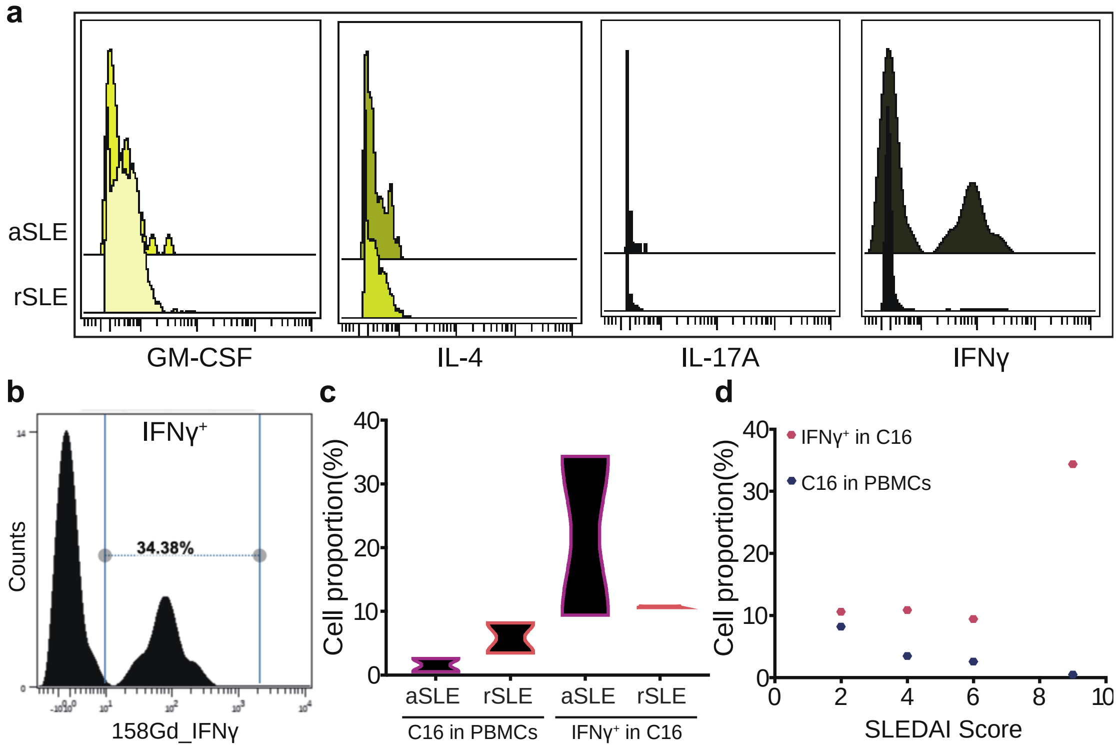

Figure S8. Cluster 16 ($CD8^{+}CD27^{+}CXCR3^{-}$ T) cells display enhanced IFNγ secretion in active SLE. PBMCs of another 4 recruited SLE patients are stimulated with the cocktail of PMA, Ionomycin and Brefeldin A, measured via CyTOF and gated manually. (A) Histograms of GM-CSF, IL-4, IL-17A and IFNγ expressions of $CD8^{+}CD27^{+}CXCR3^{-}$ T cells from representative aSLE and rSLE. (B) Manual gating strategy of $IFN\gamma^{+}$ cells in $CD8^{+}CD27^{+}CXCR3^{-}$ T cells. (C) $CD8^{+}CD27^{+}CXCR3^{-}$ T cell abundance in PBMCs and $IFN\gamma^{+}$ cell frequency in $CD8^{+}CD27^{+}CXCR3^{-}$ T cells. (D) Correlation analysis between SLEDAI scores and $CD8^{+}CD27^{+}CXCR3^{-}$ T cell

abundance in PBMCs, IFNγ^{+} cell frequency in CD8^{+}CD27^{+} CXCR3^{-} T cells. aSLE, active SLE patients; CyTOF, mass cytometry; GM-CSF, granulocyte-macrophage colony-stimulating factor; HCs, healthy controls; IFNγ, Interferon γ; IL-4, interleukin-4; IL-17A, interleukin-17A; PBMCs, peripheral blood mononuclear cells; PMA, phorbol 12-myristate 13-acetate; rSLE, remission SLE patients; SLE, systemic lupus erythematosus.

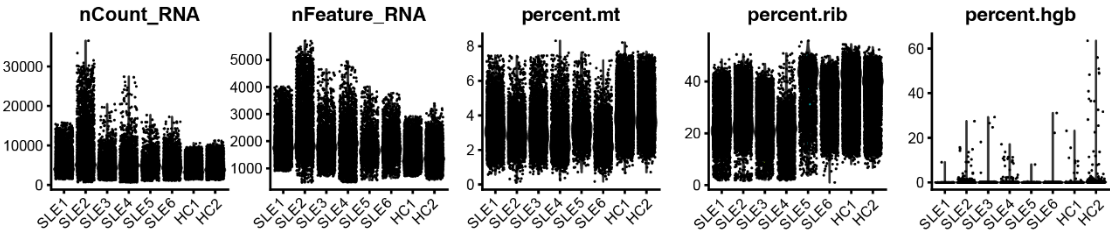

Figure S9 Detailed information on single-cell data quality and batch effects.

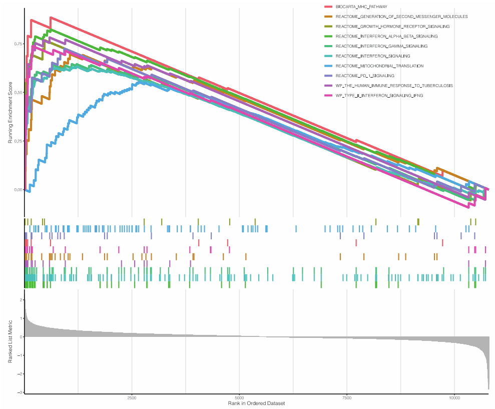

Figure S10. Based on the Gene Set Enrichment Analysis (GSEA), top 10 upregulated signaling pathway in CD8^{+}CD27^{+}CXCR3^{-} T cells in aSLE compared with HCs.

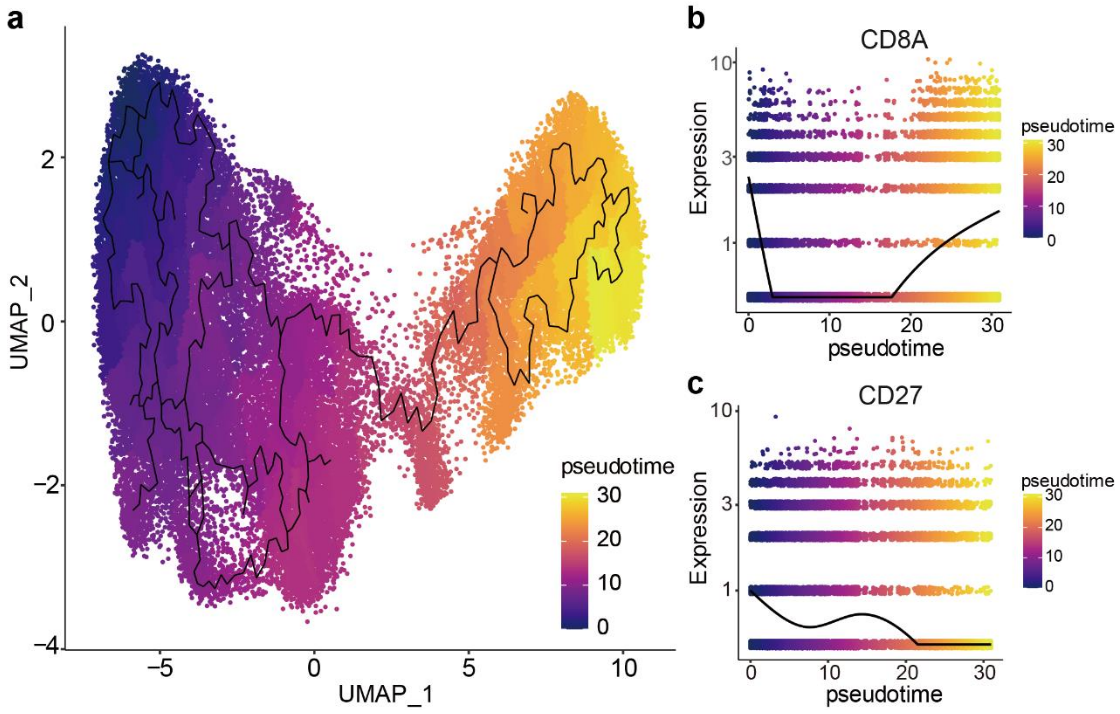

Figure S11. The pseudo-time series analysis of (A) cell subtypes, (B) CD8A and （C）CD27 biomarker expression from public scRNA-seq data.

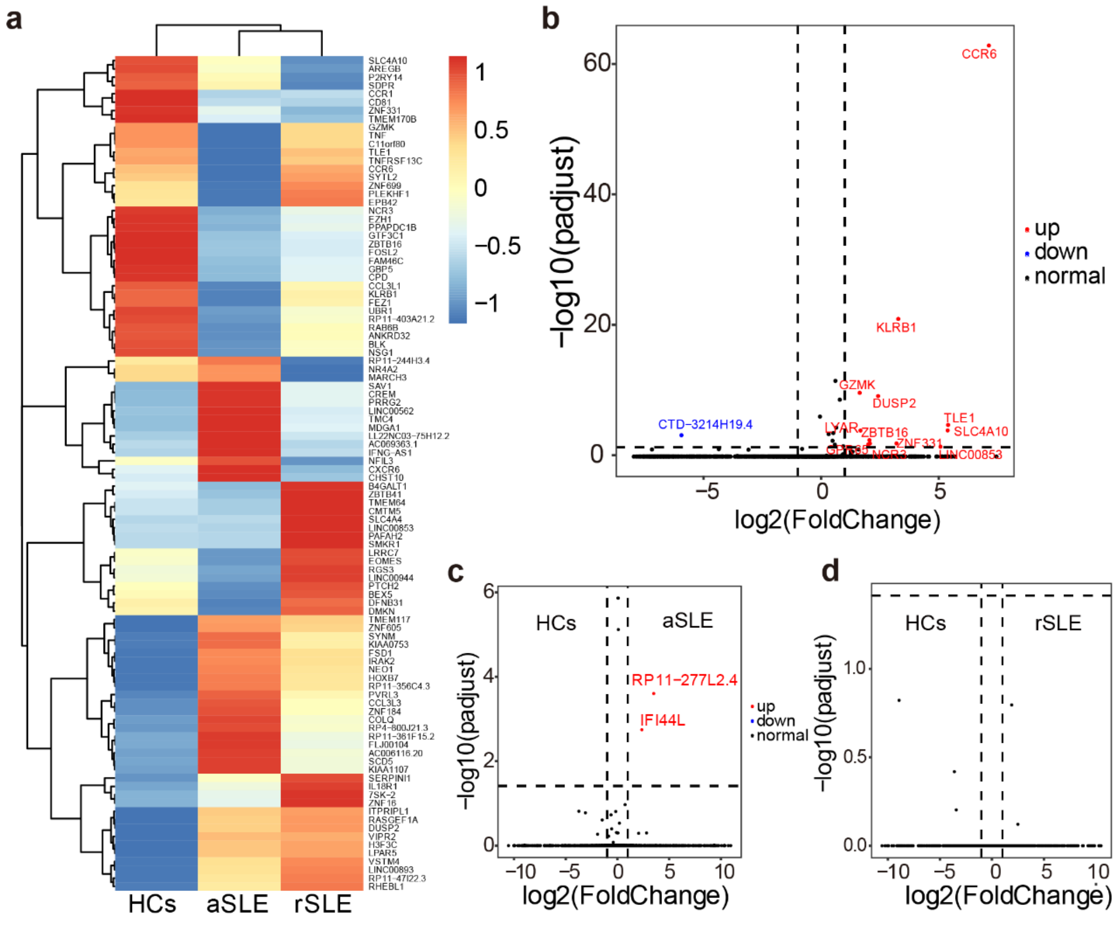

Figure S12. Gene expression results of 8 HCs, 8 aSLEs and 8 rSLEs from published data. (A) The heatmap showed the top differentially express genes (DEGs) in CD8$^+$CD27$^+$CXCR3$^-$ T cells. (B) The volcano result showed the genes which increased or decreased significantly in CD8$^+$CD27$^+$CXCR3$^-$ T cells. (C) The volcano result showed the genes which increased or decreased significantly of CD8$^+$CD27$^+$CXCR3$^-$ T cells in aSLE compared with HCs. (D) The volcano result showed the genes which increased or decreased significantly of CD8$^+$CD27$^+$CXCR3$^-$ T cells in rSLE compared with HCs.

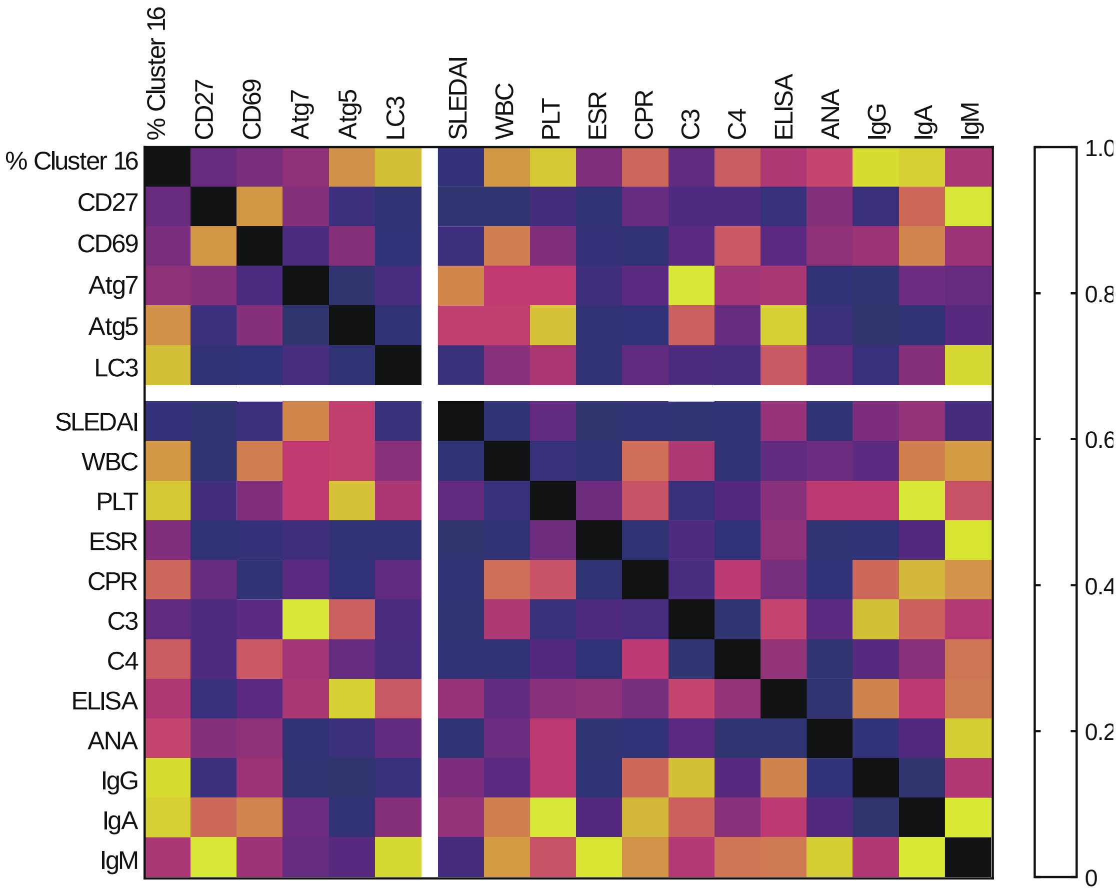

Figure S13. p values of Spearman's correlation coefficient between Clinical disease activity and molecular signatures of Cluster 16 ($CD8^{+}CD27^{+}CXCR3^{-}$ T) cells. ANA, antinuclear antibodies; C3, complement 3; C4, complement 4; CPR, C-reactive protein; ELISA, enzyme-linked immunosorbent assay; ESR, erythrocyte sedimentation rate; PLT, platelets; SLE, systemic lupus erythematosus; SLEDAI, SLE disease activity index; WBC, white blood cells.

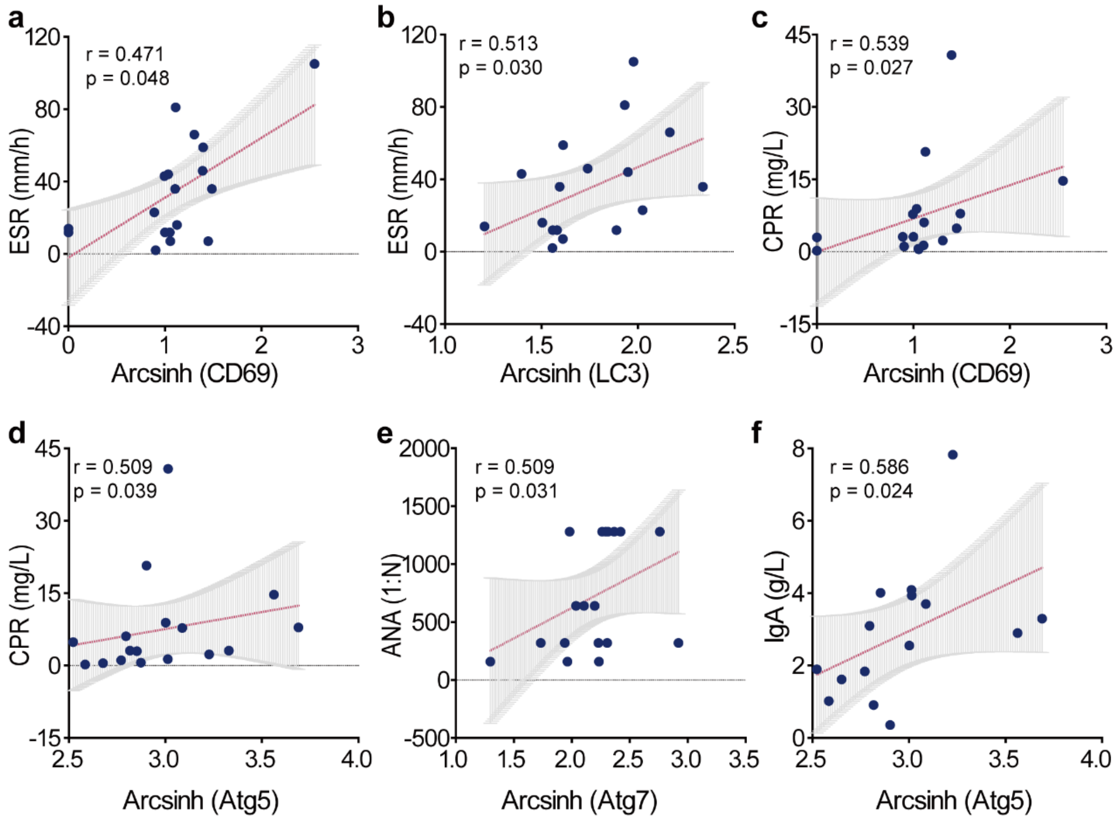

Figure S14. SLE disease activity is positively associated with the effector function and autophagy activity of Cluster 16 ($CD8^{+}CD27^{+}CXCR3^{-}$ T) cells. Correlation analysis between ESR values and (A) arcsinh (CD69) and (B) arcsinh (LC3). Correlation analysis between CPR values and (C) arcsinh (CD69) and (D) arcsinh (Atg5). (E) Correlation analysis between ANA values and arcsinh (Atg7). (F) Correlation analysis between IgA values and arcsinh (Atg5). All r and p values are based on Spearman's correlation coefficient. ANA, antinuclear antibodies; C3, complement 3; C4, complement 4; CPR, C-reactive protein; ELISA, enzyme-linked immunosorbent assay; ESR, erythrocyte sedimentation rate; PLT, platelets; SLE, systemic lupus erythematosus; SLEDAI, SLE disease activity index; WBC, white blood cells.

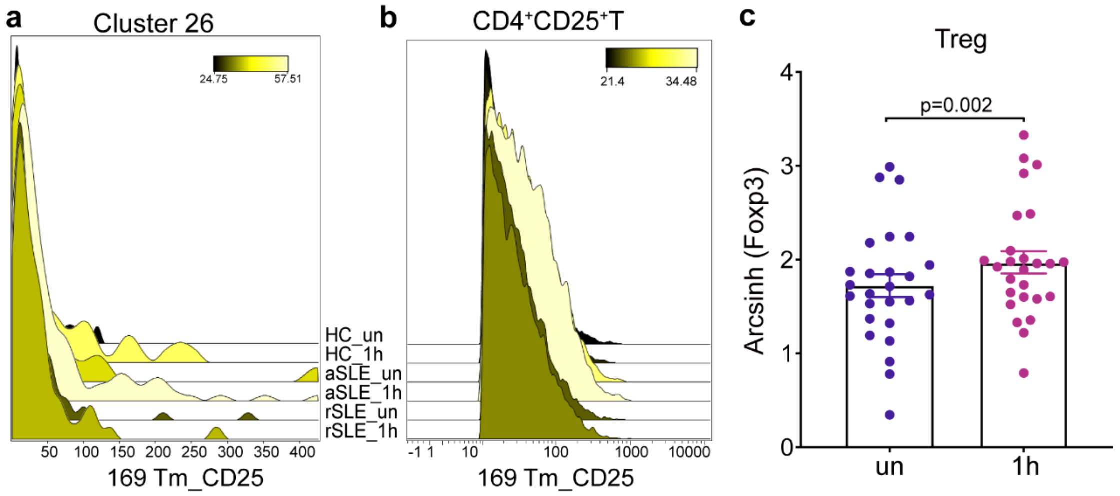

Figure S15. IL-2 treatment *in vitro* upregulates CD25 and Foxp3 expression of Treg cells. Histograms of CD25 expression of (A) Treg cells (Cluster 26) and (B) manually gated $CD4^{+}CD25^{+}$ T cells from representative samples across3 groups under different conditions. (C) Foxp3 expression of Treg cells (Cluster 26) from unstimulated and IL-2 treatment PBMCs. In the box plot, data are presented as Mean±SEM and the p value is based on two-tailed Wilcoxon matched-pairs signed rank test. aSLE, active SLE patients; HCs, healthy controls; IL-2, interleukin-2; PBMCs, peripheral blood mononuclear cells; rSLE, remission SLE patients; un, unstimulated; 1 h, PBMCs treated with IL-2 for 1 h.

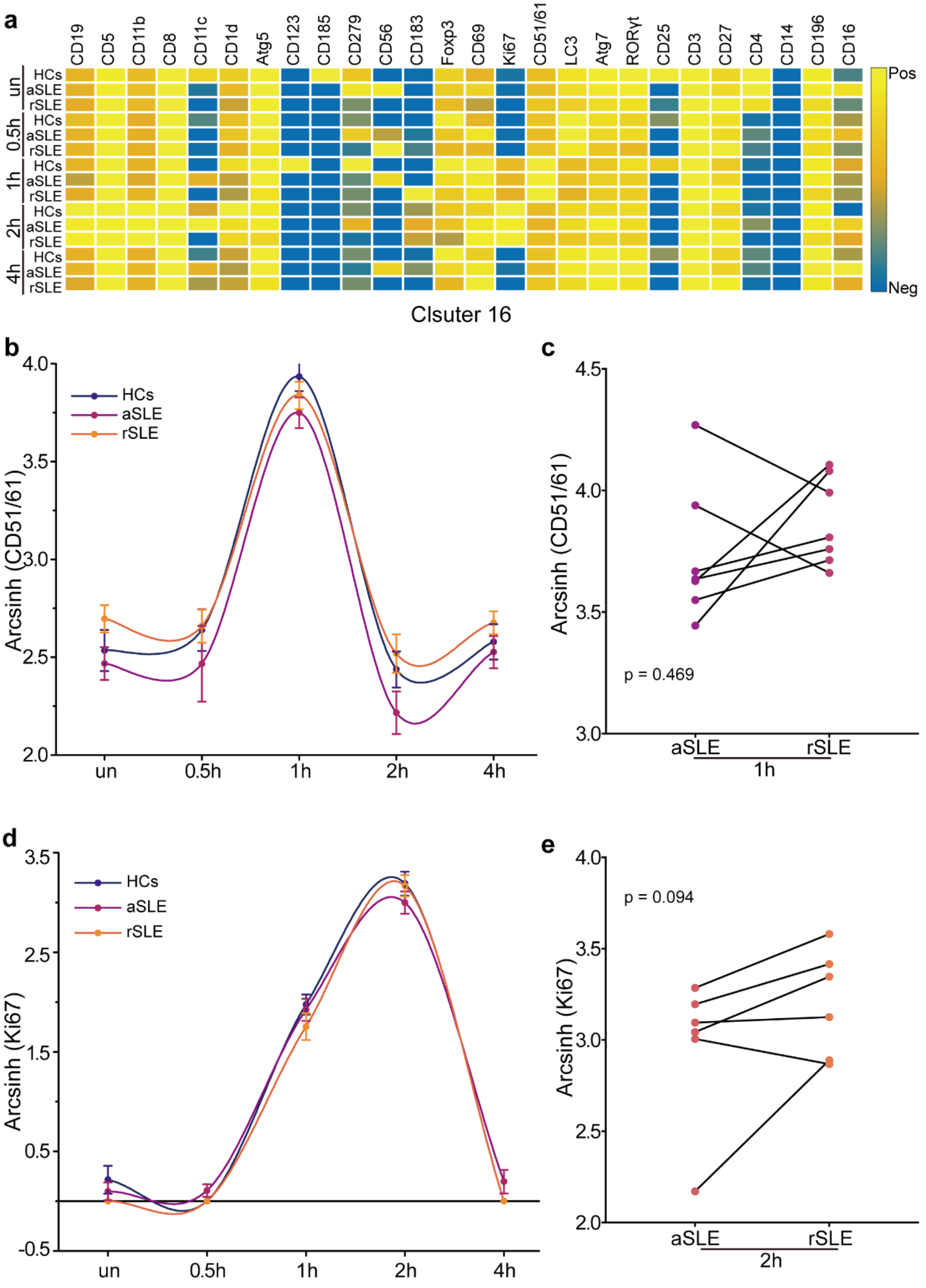

Figure S16. IL-2 treatment upregulates expressions of CD51/61 and Ki67 of Cluster 16 ($CD8^{+}CD27^{+}CXCR3^{-}$ T) cells. (A) Relative expressions of biomarker across 3 groups under 5 conditions. (B) The line chart of CD51/61 expression from

PBMCs under different IL-2 treatments. (C) The paired sample t-test of CD51/61 expression between matched treatment-naïve aSLE and rSLE after treated with IL-2 for 1h. (D) The line chart of Ki67 expression under different IL-2 treatments. (E) The paired sample t-test of Ki67 expression between matched treatment-naïve aSLE and rSLE after treated with IL-2 for 1h. For paired sample t-tests, p values are based on two-tailed Wilcoxon signed-rank tests between groups. aSLE, active SLE patients; HCs, healthy controls; IL-2, interleukin-2; PBMCs, peripheral blood mononuclear cells; rSLE, remission SLE patients; un, unstimulated; 0.5 h, PBMCs treated with IL-2 for 0.5 h; 1 h, PBMCs treated with IL-2 for 1 h; 2 h, PBMCs treated with IL-2 for 2 h; 4 h, PBMCs treated with IL-2 for 4 h.

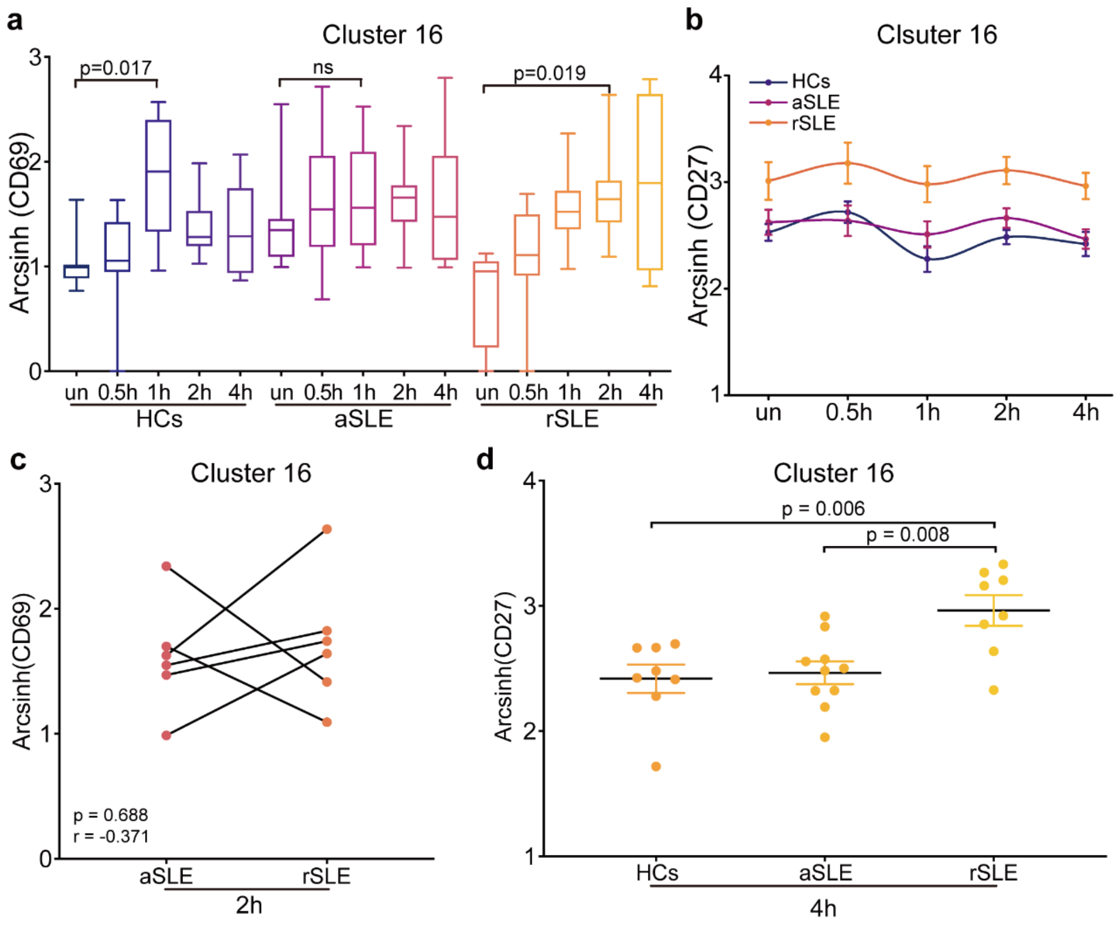

Figure S17. IL-2 treatment upregulates CD69 expression of Cluster 16 (CD8+CD27+CXCR3- T) cells. (A) Individual expressions of CD69 across 3 groups under different IL-2 treatments. The p value of HCs is based on Friedman test and p values of treatment-naïve aSLE and rSLE are based on Kruskal-Wallis test, post-test $p < 0.05$. (B) The paired sample t-test of CD69 expression between matched treatment-naïve aSLE and rSLE after treated with IL-2 for 2h. For the paired sample t-test, the p value is based on a two-tailed paired t test. (C) The line chart of arcsinh (CD27) of $CD8^+CD27^+CXCR3^-$ T cells from PBMCs under different IL-2 treatments. (D) Individual CD27 expression across 3 groups after IL-2 treatment for 4h. In the box plot, data are presented as Mean±SEM and all p values are based on Kruskal-Wallis test, post-test $p < 0.05$. aSLE, active SLE patients; HCs, healthy controls; IL-2, interleukin-2; rSLE, SLE patients in remission; 0.5 h, PBMCs treated with IL-2 for 0.5 h; 1 h, PBMCs treated with IL-2 for 1 h; 2 h, PBMCs treated with IL-2 for 2 h; 4 h, PBMCs treated with IL-2 for 4 h.

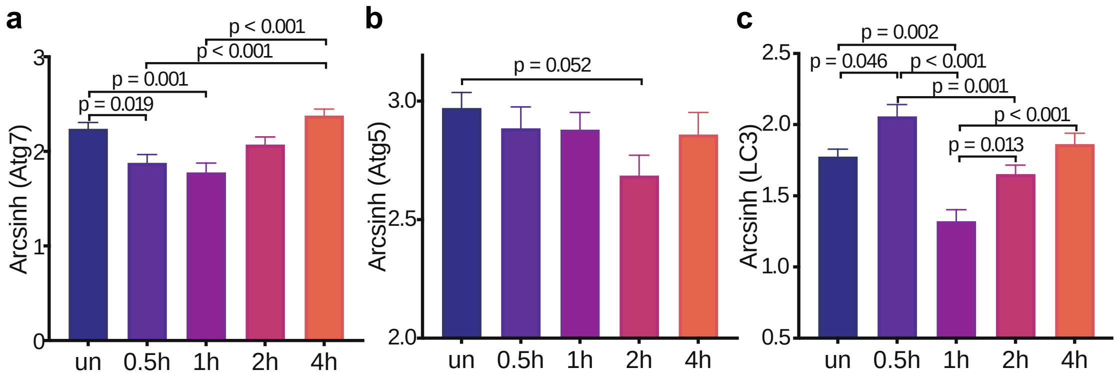

Figure S18. Expression levels of autophagy-related biomarkers of Cluster 16 ($CD8^+CD27^+CXCR3^-$ T) cells after IL-2 treatment. (A) Atg7 expressions under different IL-2 treatment conditions. (B) Atg5 expressions showed a slight decrease after

IL-2 treatment for 2 h. (C) Atg7 expressions under different IL-2 treatment conditions. In the box plot, data are presented as Mean±SEM and all p values are based on Kruskal-Wallis test, post-test $p < 0.05$. aSLE, active SLE patients; HCs, healthy controls; IL-2, interleukin-2; ns, not significant; rSLE, remission SLE patients; SEM, standard error of the mean; SLE, systemic lupus erythematosus; un, unstimulated; 0.5 h, PBMCs treated with IL-2 for 0.5 h; 1 h, PBMCs treated with IL-2 for 1 h; 2 h, PBMCs treated with IL-2 for 2 h; 4 h, PBMCs treated with IL-2 for 4 h.

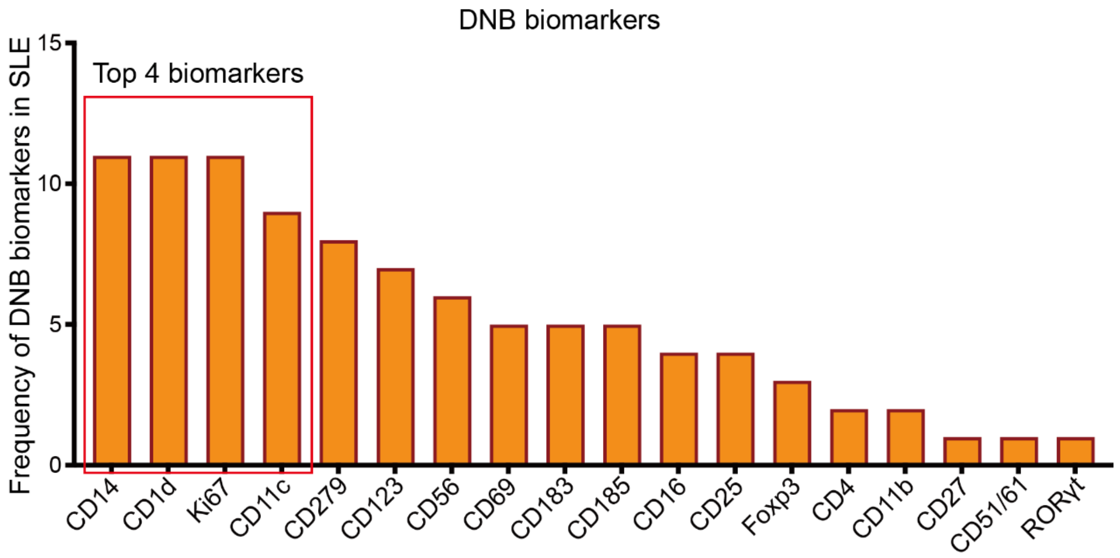

Figure S19. The frequency of each DNB biomarker in SLE patients.

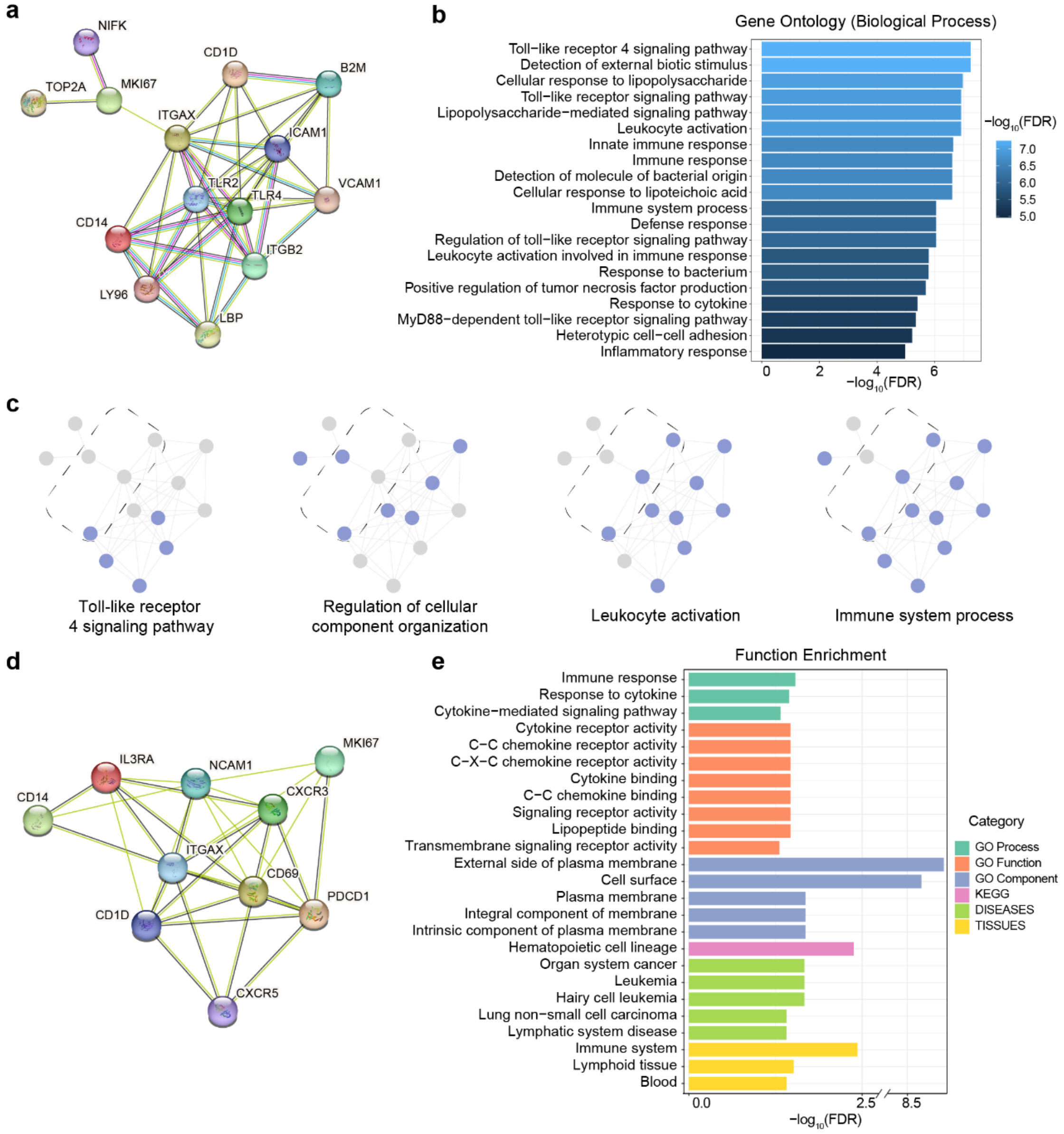

Figure S20. DNB members are involved in immune system processes (A) Association network of DNB core proteins and their first-order neighbors. The PPI enrichment *p value* of four DNB core proteins is 0.00219, including *CD14*, *CD1d* (*CD1D*), *Ki67* (*MKI67*) and *CD11c* (*ITGAX*). (**B**) Bar plot showing the GO enrichment of DNB core proteins and their first-order neighbors. (**C**) The network diagram showing the involvement of proteins in four GO terms, which include *Toll-like receptor 4 signaling pathway*, *Regulation of cellular component organization*, *Leukocyte activation* and *Immune system process*. Proteins in the pathway are labeled in purple, while proteins not involved in the pathway are labeled in gray. The points in the dashed box represent four DNB core proteins. (**D**) Association network of DNB proteins with

a frequency of greater than or equal to 5 in SLE patients. The PPI enrichment *p value* of ten DNB proteins: < 1.0e-16, including *CD14*, *CD1d* (*CD1D*), *Ki67* (*MKI67*), *CD11c* (*ITGAX*), *CD279* (*PDCD1*), *CD123* (*IL3RA*), *CD56* (*NCAM1*), *CD69*, *CD183*(*CXCR3*) and *CD185* (*CXCR5*). (**E**) Bar plot showing the function enrichment of ten DNB proteins.

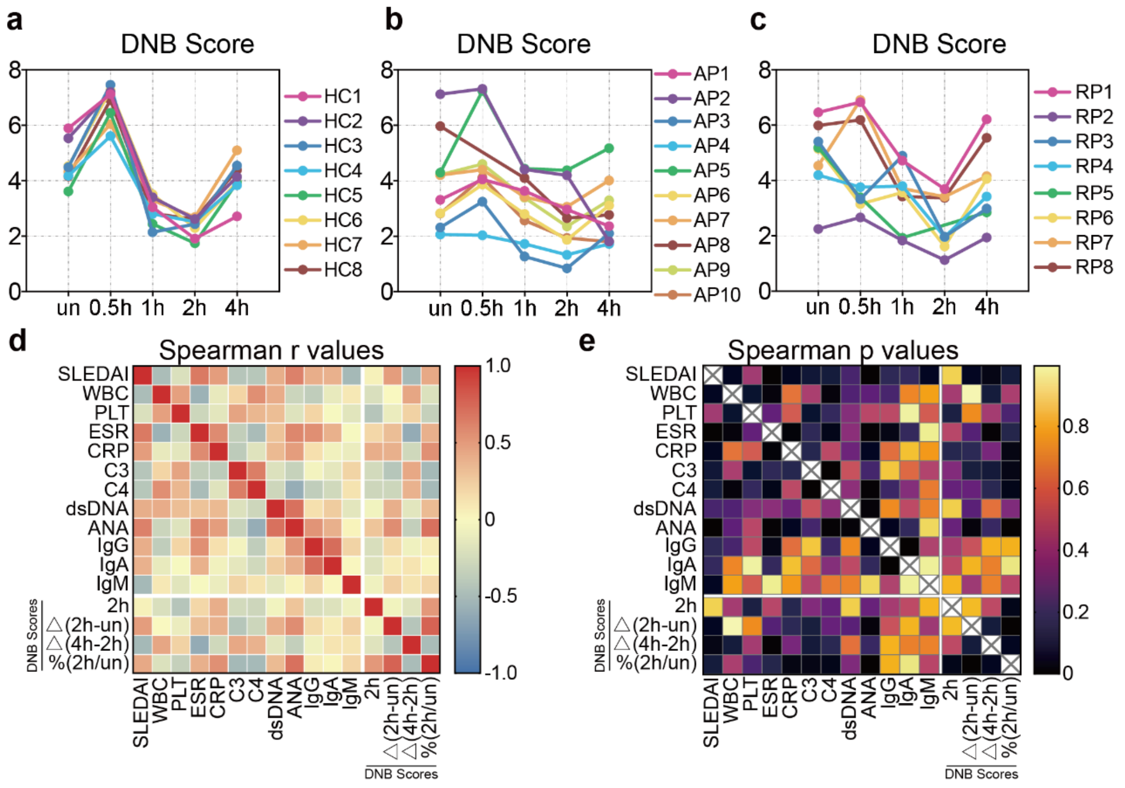

Figure S21. DNB scores are highly correlated with clinical features in SLE. (A-C) The individual DNB score of (A) HCs, (B)aSLE and (C) rSLE. (D, E) The (D) r and (E) p values of Spearman's correlation coefficient between Clinical disease activity and DNB scores. DNB, dynamic network biomarkers; ESR, erythrocyte sedimentation rate; SLEDAI, SLE disease activity index.

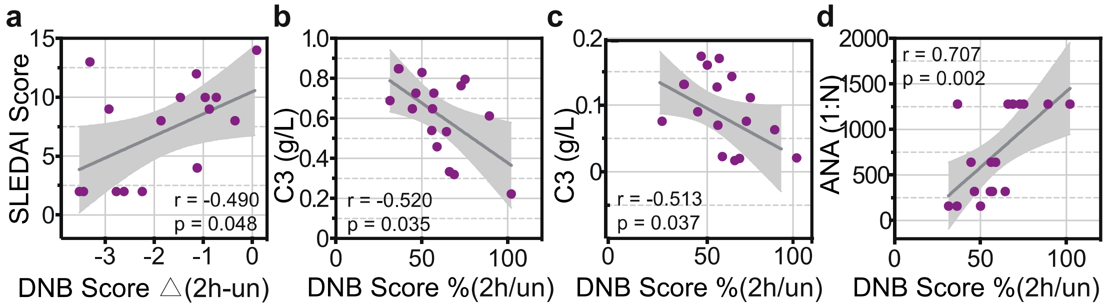

Figure S22. 2h treatment related DNB scores are highly correlated with clinical features in SLE. (A) The difference between 2h IL-2 treatment and unstimulated situation were positively correlated with SLEDAI score. (B-D) The ratio between 2h IL-2 treatment and unstimulated conditions were negatively correlated with (B) C3 and (C) C4 while (D) positively correlated with ANA. DNB, dynamic network biomarkers; ESR, erythrocyte sedimentation rate; SLEDAI, SLE disease activity index.
